# Single-cell image analysis reveals over-expression of organic anion transporting polypeptides (OATPs) in human glioblastoma tissue

**DOI:** 10.1093/noajnl/vdac166

**Published:** 2022-10-14

**Authors:** Elizabeth Cooper, Zoe Woolf, Molly E V Swanson, Jason Correia, Patrick Schweder, Edward Mee, Peter Heppner, Clinton Turner, Richard L M Faull, Emma L Scotter, William A Denny, Peter J Choi, Mike Dragunow, Jiney Jose, Thomas I-H Park

**Affiliations:** Department of Pharmacology, University of Auckland, Auckland, New Zealand; Neurosurgical Research Unit, The Centre for Brain Research, University of Auckland, Auckland, New Zealand; Auckland Cancer Society Research Centre, University of Auckland, Auckland, New Zealand; The Hugh Green Biobank, University of Auckland, Auckland, New Zealand; Department of Pharmacology, University of Auckland, Auckland, New Zealand; Neurosurgical Research Unit, The Centre for Brain Research, University of Auckland, Auckland, New Zealand; Department of Biological Sciences, University of Auckland, Auckland, New Zealand; Neurosurgical Research Unit, The Centre for Brain Research, University of Auckland, Auckland, New Zealand; Department of Neurosurgery, Auckland City Hospital, Auckland, New Zealand; Neurosurgical Research Unit, The Centre for Brain Research, University of Auckland, Auckland, New Zealand; Department of Neurosurgery, Auckland City Hospital, Auckland, New Zealand; Neurosurgical Research Unit, The Centre for Brain Research, University of Auckland, Auckland, New Zealand; Department of Neurosurgery, Auckland City Hospital, Auckland, New Zealand; Department of Neurosurgery, Auckland City Hospital, Auckland, New Zealand; Neurosurgical Research Unit, The Centre for Brain Research, University of Auckland, Auckland, New Zealand; Department of Anatomical Pathology, LabPlus, Auckland City Hospital, Auckland, New Zealand; Neurosurgical Research Unit, The Centre for Brain Research, University of Auckland, Auckland, New Zealand; Department of Biological Sciences, University of Auckland, Auckland, New Zealand; Auckland Cancer Society Research Centre, University of Auckland, Auckland, New Zealand; Auckland Cancer Society Research Centre, University of Auckland, Auckland, New Zealand; Department of Pharmacology, University of Auckland, Auckland, New Zealand; Neurosurgical Research Unit, The Centre for Brain Research, University of Auckland, Auckland, New Zealand; The Hugh Green Biobank, University of Auckland, Auckland, New Zealand; Auckland Cancer Society Research Centre, University of Auckland, Auckland, New Zealand; Department of Pharmacology, University of Auckland, Auckland, New Zealand; Neurosurgical Research Unit, The Centre for Brain Research, University of Auckland, Auckland, New Zealand; The Hugh Green Biobank, University of Auckland, Auckland, New Zealand

**Keywords:** glioblastoma, organic anion transporting polypeptides, OATP, tumor microenvironment

## Abstract

**Background:**

Glioblastoma (GBM) is the most common and aggressive primary brain tumor in adults. Whilst the role of the efflux transporters are well established in GBM, the expression and function of uptake transporters, such as the organic anion transporting polypeptide (OATP) family, are not well understood. OATPs possess broad substrate specificity that includes anti-cancer agents; therefore, we sought to investigate the expression of four OATP isoforms in human GBM cell types using patient tumor tissue.

**Methods:**

We used fluorescent immunohistochemical labeling of paraffin-embedded surgically resected tissues and single-cell image analysis methods to explore the expression of the OATP isoforms in different tumor cell types through co-labeling with cell-type specific markers, such as IBA1 (pan-myeloid), GFAP (tumor cell), PDGFRβ (stromal cell), and UEA-1-lectin (endothelial).

**Results:**

We found significant over-expression of all the OATP isoforms (OATP1A2, 2B1, 1C1 and 4A1) in GBM tumor sections when compared to non-neoplastic brain. A single-cell image analysis revealed that OATPs were significantly upregulated throughout the tumor parenchyma, with significantly higher expression found on lectin-positive blood vessels and IBA1-positive myeloid cells in GBM compared to non-tumor brain tissue. Qualitative analysis of the four OATP isoforms demonstrated greater expression of OATP4A1 in peri-necrotic regions of GBM tissue, which correlated with hypoxia-related markers within the Ivy GAP RNAseq dataset.

**Conclusion:**

Here, we demonstrate, for the first time, the protein expression of four OATPs in human GBM tissue, including upregulation within the tumor microenvironment by myeloid cells and tumor vasculature, and isoform-specific upregulation within hypoxic niches.

**Key Points**

- Protein quantification of tumor-associated OATP isoforms in GBM and normal brain tissues.
- Localized the expression of OATP isoforms to GBM tumor-associated cell types.
- Demonstrated that tumor microenvironments can influence OATP expression.

**Importance of the Study**

Organic anion transporting polypeptides (OATPs) are critical solute carrier proteins implicated in tumor cell metabolism and drug-delivery. RNA-sequencing studies have shown that GBM tumors upregulate four OATP isoforms, yet, no study has elucidated their protein expression and localization in patient GBM tissues. This study demonstrates for the first time that these OATP isoforms are all highly expressed in GBM compared to non-neoplastic brains and can be influenced by tumor microenvironments. These are crucial information that will enable researchers interested in GBM metabolism and drug-delivery to utilize OATPs as a biological and therapeutic target to understand and modify disease progression.

Glioblastoma (GBM) is the most common and aggressive primary central nervous system (CNS) neoplasm, with a median survival of only 12–15 months.^1^ The most common multimodal treatment option includes surgical resection, followed by concurrent radiation and temozolomide (TMZ) therapy. Despite numerous advances in our understanding of GBM tumorigenesis, this has not translated to significant advancements in therapeutics, where median patient survival time has only increased by a further three months.^1^ Treatment efficacy is challenged by the protection of tumor cells from chemotherapeutic agents by the blood–brain barrier (BBB).^2^ While a multiplicity of transporters ensure a wide range of molecules are efficiently extruded back into the blood, GBM tumors are highly dependent on the selective entry of certain compounds through specialized uptake transporters at both the blood-tumor barrier and within the tumor itself.^3^ Accumulating evidence supports the idea that solute carrier transporters, including organic anion transporting polypeptides (OATPs), may play an active role in the influx of nutrients into tumor cells, although their expression in GBM is yet to be established.^4,5^ In addition, we and others have identified a potential role for OATPs in the uptake of anti-cancer therapies in GBM.^6–9^ Therefore, characterizing the expression of these transporters in GBM could identify molecular targets and facilitate the delivery of effective therapeutics into the tumor parenchyma.

Indeed, pharmacologically relevant uptake transporters are found within the solute carrier organic anion (SLCO) family. The OATP family represent a major subfamily of solute carriers, and subsequently are expressed throughout many tissues within the human body.^10^ Eleven human OATPs are currently known with shared structure and substrate specificity, with the OATP1 family being the most well characterized.^4^ The OATP1 family includes four members; OATP1A2, 1B1, 1B3, and 1C1, which display broad and overlapping substrate specificity.^11,12^ The OATP2 family includes OATP2A1 and OATP2B1, both of which display relatively narrow substrate specificity compared to other OATPs. The OATP4 family includes 4A1 and 4C1. The OATP3, OATP5, and OATP6 families include OATP3A1, 5A1, and 6A1, respectively.^11,12^ The broad range of structure and substrate specificities of each OATP family underpins OATP’s collective function as multi-specific *trans*-membrane carriers that can transport a structurally diverse array of endogenous and xenobiotic compounds.^11,12^ Considering their broad substrate specificity, OATPs could be an effective means for the targeted transport of therapeutic drugs.

OATPs are hypothesized to be responsible for the uptake of steroid hormones, as well as opioids (OATP1A2) and statins (OATP1A2 and 2B1), into the brain.^13–16^ However, the low expression of OATPs in the human brain has challenged a thorough characterization of their function in the brain and BBB.^17,18^ Whilst OATP RNA and protein expression are low in the brain relative to other normal non-malignant tissues, the expression of OATPs can be altered in disease conditions, including cancer.^10,19^ Indeed, levels of OATPs are increased in multiple malignancies tissues, and are summarized in Supplementary Table S1. Importantly, there is a long list of anti-cancer agents reported to be substrates of OATPs; therefore, it is presumed that OATPs may play a significant role in the disposition of substrates in cancer therapy, including imatinib, paclitaxel and methotrexate.^4,19,20^

In GBM, bulk RNA-sequencing has revealed the transcriptional expression of multiple OATP subtypes within the tumor parenchyma.^21,22^ Specifically, Bronger et al^21^ investigated the mRNA expression of ABCC drug efflux pumps and OATPs in human gliomas and the blood–tumor barrier, revealing the expression of *SLCO1A2, 1C1, 2B1* and *4A1* in human glioma samples. However, no study to date has provided a methodical quantification of OATP protein expression that supplements published gene expression data for GBM. Given the relatively low expression of OATPs within the normal human brain, in addition to the evidence of increased OATP expression in malignant tissue, we sought to investigate the protein expression pattern of OATP subtypes in human GBM tissue using single-cell analysis. The identification of cell-type specific, and tumor microenvironment-dependent expression of OATPs in human GBM tissue offers the possibility of tumor-specific targeting of anti-cancer agents.

## Methods

### Human Tissue Selection

Human brain tissue was obtained with written patient consent (Northern X Ethics Committee and the University of Auckland Human Participants Ethics Committee) from surgical resection of grade IV tumors or epilepsy surgeries conducted at Auckland City Hospital (Supplementary Tables S2 and S3). All epilepsy tissues were obtained from temporal lobectomy surgeries for refractory mesial temporal epilepsy.

#### Fresh-frozen neurosurgical GBM tumor and epilepsy specimens for RT-qPCR.

—Fresh-frozen neurosurgical tissue from two epilepsy (non-tumor) and eight GBM cases were used for the characterization of the gene expression profiles of each of these transporters through Real-time quantitative reverse transcriptase polymerase chain reaction (RT-qPCR). The cases used are detailed in Supplementary Table S3. RNA extraction from fresh-frozen tissue was performed using the ReliaPrep^TM^ RNA Miniprep Systems (Promega) as previously described.^23^

#### Paraffin-embedded GBM tumor and epilepsy middle temporal gyrus for immunohistochemistry.

—Fresh biopsy tissues were fixed in 15% formalin in 0.1 M phosphate buffer before being paraffin-embedded as previously described.^24–26^ For immunohistochemical studies, 7-μm thick sections from GBM (*n* = 25 cases) and epilepsy cases (*n* = 8 cases) were used (Supplementary Table S2). GBM tumors were classified according to the 2016 WHO classification of CNS tumors, where 24 cases were classified as GBM, IDH-wild type, and one was classified as GBM, IDH mutant.^27^ Male to female ratio (GBM = 17:8, Epilepsy = 3:5) are reflective of the expected ratios for patients diagnosed with GBM.^28,29^ For GBM patients, the ratio of unmethylated *MGMT* promoters was consistent with that reported elsewhere (3:2).^30,31^ The mean age of the GBM tumor and epilepsy cases were 60 ± 9 years and 36 ± 9 years, respectively.

#### Immunohistochemical characterization of the expression of organic anion transporting polypeptides in human GBM tissue.

—Fluorescent immunohistochemistry was conducted as previously described^24,32^ in order to quantify the expression of OATPs in human GBM tissue. Serial sections were labeled with each combination within each case and imaged using a 20× (0.9 NA) objective on the Metasystems VSlide slide scanner (Mestasystems), running Metafer software (V.3.12.1). The regions of interest were selected and subsequently stitched within the automated MetaCyte software, as previously described.^24^ Acquired images were opened and extracted using the VSViewer v2.1.112. Single-stain controls were performed during a pilot run to ensure that our multiplex immunohistochemistry with the appropriate filters. No-primary controls were included for each combination, in both tumor and non-tumor tissue; representative images are seen in Supplementary Figure S1.

### Metamorph Custom Image Analysis Pipelines

The overall image analysis pipeline is shown in Supplementary Figures S2 and S3. For the quantification of OATP expression in immunohistochemically labeled epilepsy and GBM tissue, we developed custom image analysis pipelines using Metamorph software (Molecular Devices), like those previously described.^24–26^ Firstly, this sought to quantify the tissue-wide expression of each OATP, as well as pan-tumor cell marker, GFAP, pan-myeloid marker, IBA1, endothelial marker, UAE-1-lectin, and stromal cell marker, PDGFRβ (Table 1). We subsequently sought to quantify the single-cell expression of OATP1A2, 2B1, 1C1, and 4A1 within myeloid cells, blood vessels, and stromal PDGFRβ-positive cells. The analysis of the expression of each MOI within each cell-type is described below, and can be visualized by the workflow in Supplementary Figure S3.

**Table 1.** Primary antibodies and visualization methods used for cell type classification in human GBM and epilepsy tissue

Target	Primary antibody	Company, catalogue number	Immunofluorescent visualization
Myeloid cells	Chicken anti-IBA1	Synaptic Systems, 234-009	IRDye^®^ 800W secondary
Endothelia	Biotinylated Lectin from *UEA-1*	Sigma Aldrich, L8262-2MG	AlexaFluor^®^ 647 streptavidin
Pericytes	Goat anti-PDGFRβ	R&D Systems, Inc., AF385	AlexaFluor^®^ 594 secondary
GBM parenchyma/astrocytes	Mouse anti-GFAP	Sigma Aldrich, G3893	AlexaFluor^®^ 546 secondary
OATPs	Rabbit anti-OATP1A2	Abcam, Ab221804	AlexaFluor^®^ 488 secondary
	Rabbit anti-OATP2B1	Thermo Fisher, PA5-42453	AlexaFluor^®^ 488 secondary
	Rabbit anti-OATP1C1	Abcam, Ab234729	AlexaFluor^®^ 488 secondary
	Rabbit anti-OATP4A1	Sigma Aldrich, HPA030669	AlexaFluor^®^ 488 secondary

### Validation of Organic Anion Transporting Polypeptide Expression Through the IvyGAP RNAseq Data Set

Gene expression within the hypoxic regions were determined by the differential expression analysis of data from Ivy GBM Atlas Project, where we compared the cellular tumor (CT) and peri-necrotic (PNZ) and pseudo-palisading cells around necrosis (PPN). Differentially expressed genes (DEG) were determined in R software v4.0.3 (R Core Team, 2020), the edgeR (v3.14), limma (v3.14), Glimma (v3.14), org.Hs.eg.db (v3.14) (45), gplots (v3.1.1) and NMF (v0.23.0) packages. DEGs were selected using a cut-off based on a significant difference in the expression (false discovery rate < 0.01, *P* < .1, Benjamini–Hochberg corrected). The collection of the data from the Ivy GAP was compliant with all applicable laws, regulations, and policies for the protection of human subjects.

### Statistical Analysis

Statistical analysis was performed using GraphPad Prism 9 (GraphPad Software Inc.) or R Software. The normality and variance were tested using the Shapiro–Wilk normality test and *F*-test of equal variance, respectively. Mann–Whitney *U*-tests were subsequently carried out accordingly when comparing the two groups. For the comparison of grouped data, two-way analysis of variances with either Sidak’s or Tukey’s multiple comparison tests were carried out. For the correlation of two or more groups, Spearman correlation matrices were performed. All assumptions were tested. Statistical significance was set at *P* ≤ .05. All displayed OATP data analyses were pooled from co-labeling with each cell-specific marker, with the average of repeated cases used for all statistical analysis and data presentation.

## Results

### OATP Isoform Expression is Increased in Human GBM Tissue at the Protein and mRNA Level

**Figure 1.**
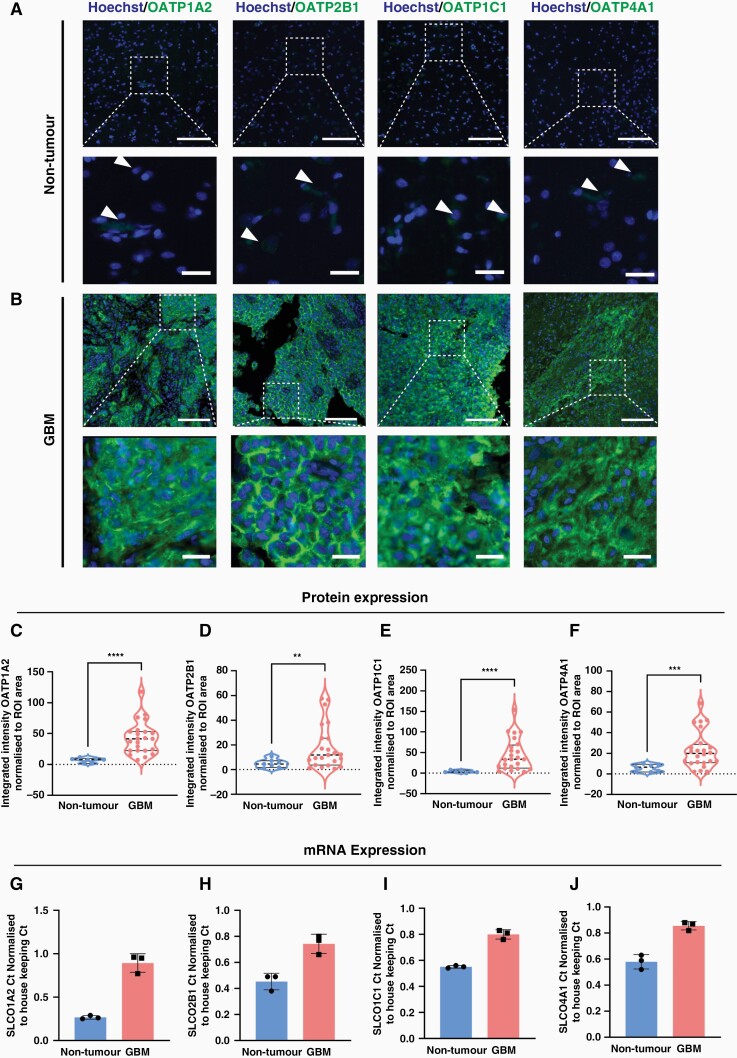
Tissue-wide integrated intensity analysis reveals an overexpression of OATP1A2, 2B1, 1C1 and 4A1 in GBM tissue. Representative immunohistochemical labelling of OATP1A2/OATP2B1/OATP1C1/OATP4A1, as labelled with Hoechst nuclear counterstain in non-tumour (A) and GBM (B) tissue; scale bar = 100 μm, inset = 75 μm. The mean integrated intensity of OATP1A2, 2B1, 1C1, and 4A1 was compared between GBM and non-tumour with a non-parametric Mann-Whitney test (C–F), data compared between tumour (*n* = 25 cases) and non-tumour (n = 8 cases) tissue with Mann-Whitney test, presented as violin plot, median ± upper and lower quartiles. qRT-PCR quantifying the mRNA expression of OATP1A2, 2B1, 1C1 and 4A1 in GBM tumour tissue compared to non-tumour MTG brain tissue (G–J), data represents the mean ± SD of GBM (n = 3 cases) and non-tumour (n = 3 cases) fresh-frozen cases. **P < 0.01, ***P < 0.001, ****P < 0.0001.

We first investigated whether the expression of OATP isoforms is altered in human GBM tissue. Immunohistochemical labeling and quantification of the integrated intensities of four OATP isoforms (OAT1A2, 2B1, 1C1 and 4A1) were carried out in both GBM and non-tumor MTG brain tissue. Quantification of the tissue-wide expression of each OATP isoform protein revealed a significant increase in the mean integrated intensity of OATP1A2 (6.1-fold, *P* < .0001), OATP2B1 (3.7-fold, *P* = .0106), OATP1C1 (12-fold, *P* = .0001), and OATP4A1 (4-fold, *P* = .0005) in GBM tumor tissue compared to non-tumor tissue (Figure 1A). Percentage area coverage of OATP expression significantly correlated with mean integrated intensity measures present in Figure 1 (Supplementary Figure S4). To complement the protein quantification, we compared the gene expression of OATPs in GBM tumor tissue (Figure 1G–J) and patient-derived GBM cells (Supplementary Figure S5A) relative to non-tumor controls. Indeed, there was a clear shift in the Ct values of *SLCO1A2, 2B1, 1C1,* and *4A1* in fresh-frozen tumor tissue when compared with non-tumor tissue. We found increased gene expression of *SLCO1A2, 2B1, 1C1,* and *4A1*, with negligible *4A1* expression detected in non-tumor tissue (Figure 1G–J). This trend was maintained *in vitro* when patient-derived GBM cells were compared with non-tumor primary brain stromal cells (Supplementary Figure S5A). Furthermore, we demonstrated that OATPs were transcriptionally regulated by HIF1-alpha *in vitro* (Supplementary Figure S5B). 3, 4-DHB (100 μM) was used to stabilize HIF1-alpha through inhibition of prolyl 4-hydroxylase. Treatment of patient-derived GBM cells with 3,4-DHB (Supplementary Figure S5) and exposure to physiological hypoxia (4 h) increased the transcription and protein expression of OATPs *in vitro* (Supplementary Figure S5C–G).

### OATP Expression Correlates With that of GFAP, PDGFRβ and IBA1 in GBM, but not in Non-tumor Tissue

**Figure 2.**
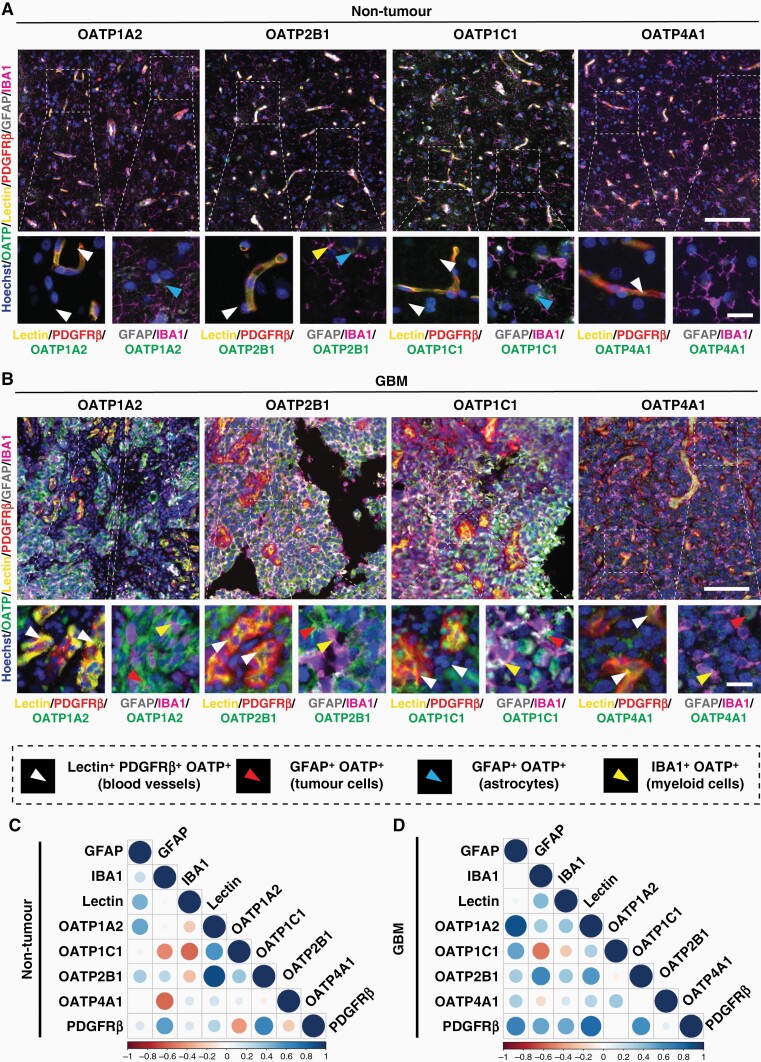
Increased expression of OATP1A2, 2B1, 1C1 and 4A1 in GBM tissue across multiple cell types. Representative immunohistochemical labelling of OATP1A2/OATP2B1/OATP1C1/OATP4A1, as labelled with pan myeloid marker, IBA1; tumour cell/astrocyte marker, GFAP; endothelial cell marker, UAE-1 lectin; stromal cell marker, PDGFRβ; and Hoechst nuclear counterstain in non-tumour (A) and GBM (B) tissue; scale bar = 100 μm, inset= 20 μm. Manual cell population classification of blood vessels, tumour cells, astrocytes, and myeloid cells from cell-specific marker expression is defined in the figure key. OATP integrated intensity normalised to ROI area was correlated with cell-type specific markers to predict cell-type specific expression of OATPs in non-tumour (C) and GBM (D) tissue using Spearman correlations. Data presented as a matrix of correlation coefficients between markers; *n* = 8 cases (non-tumour), *n* = 25 cases (GBM).

We next investigated the cell-type specific expression of each of the OATP isoforms. We utilized the pan-myeloid marker, IBA1; pan astrocyte (non-tumor) and tumor cell marker GFAP; endothelial cell marker (UAE-1-lectin); and stromal cell marker PDGFRβ. We observed that expression of OATP1A2, 2B1, 1C1 and 4A1 was predominantly confined by the vasculature in non-tumor tissue, with sparse immunoreactivity by non-vascular cells (Figure 2A). Comparatively, OATP expression appeared widespread in GBM tissue, with immunoreactivity observed not only on vascular structures, but by IBA1^+^ cells, and GFAP^+^ cells throughout the tumor parenchyma (Figure 2B). Specifically, OATP1A2 and OATP2B1 were expressed by IBA1^+^ cells and blood vessels within the tumor (Figure 2B). Interestingly, we identified IBA1^+^OATP^+^ cells within GBM tumor tissue for all four OATP isoforms, in addition to OATP^+^ blood vessel immunoreactivity (Figure 2D). OATP^+^ blood vessel immunoreactivity was also observed for all four OATP isoforms, although this was much lower in comparison to the GBM tumor expression (Figure 2A, B). We also identified weak immunoreactivity of OATP1A2, 2B1 and 1C1 on GFAP^+^ cells in non-tumor tissue (Figure 2B).

Following these observations, we correlated the average integrated intensity (normalized to ROI area) of each OATP isoform with cell-type specific markers in GBM and non-tumor cases to determine if OATP expression was preferentially driven by a specific cell population. Indeed, IHC analysis revealed distinct correlation profiles between GBM tissue and non-tumor tissue (Figure 2C, D). In GBM tissue, OATP1A2 had a strong positive correlation with GFAP (*r* = 0.8584, *P* < .00001) and PDGFRβ (*r* = 0.8341, *P* < .00001), and a moderate positive correlation with IBA1 (*r* = 0.5590, *P* = .0056), indicating OATP1A2 expression by astrocytes, PDGFRβ ^+^ stromal cells and myeloid cells, respectively. OATP2B1 had a moderate positive correlation with IBA1 (*r* = 0.7341, *P* = .0001) and PDGFRβ (*r* = 0.6812, *P* = .0003), indicating OATP2B1 expression by myeloid cells and PDGFRβ ^+^ stromal cells, respectively. Comparatively, OATP1C1 had a significant weak positive correlation with GFAP (*r* = 0.4703, *P* = .0235), and no significant correlation with any other MOIs. OATP4A1 did not significantly correlate with any of the MOIs. In contrast, there was no statistically significant correlation between the four OATP isoforms and the MOIs in non-tumor tissue.

### Single-Cell Analysis of OATP Expression Reveals Increased Expression on Myeloid Cells and Vasculature Cells in GBM Tissue

**Figure 3.**
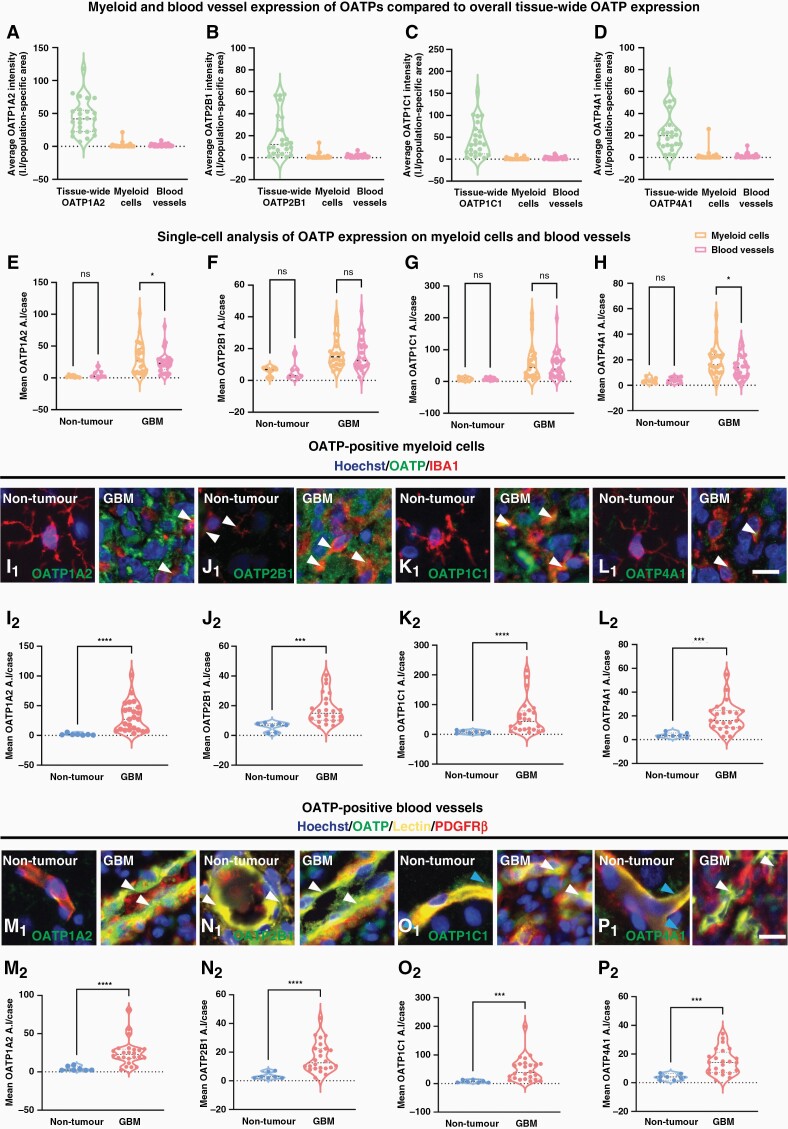
Single-cell average intensity analysis of the cell-type specific expression of OATP1A2, 2B1, 1C1 and 4A1 revealed increased expression on myeloid cells and vasculature in GBM tissue. The tissue-wide expression of OATP1A2 (A), 2B1 (B), 1C1 (C), and 4A1 (D) was compared with single-cell analysis of the average OATP intensity per cell within the OATP-positive—IBA1, and lectin masks in GBM tissue. Single-cell analysis of the mean average intensity per case of each OATP isoform within OATP-positive—IBA1, and lectin masks in tumor and non-tumor tissue (E–H). Pooled single-cell analysis of the mean stain average intensity per cell was compared for each OATP isoform within the IBA1-positive (I–L) and lectin-positive mask (M–P) in GBM and non-tumor tissue. Each of the OATP isoforms was individually compared between GBM and non-tumor tissue with a Mann–Whitney test, data presented as median ± upper and lower quartiles (I–P). Representative images of OATP isoforms in GBM and non-tumor IBA1-positive cells and blood vessels (white arrow), blue arrow denotes vascular associated OATP immunoreactivity; scale bar = 10 μm. Significance was determined using an unpaired Mann–Whitney test. *n* = 8 cases (non-tumor) *n* = 25 cases (GBM); *P < .05, ***P < .001, ****P < .0001.

Tissue-wide analysis revealed an over-expression of each OATP isoform in GBM tumor tissue (Figures 1 and 2). Spearman correlation matrices further suggested that there may be isoform-dependent expression by specific cell-types within the tumor parenchyma (Figure 2C, D). Therefore, we investigated whether the increased OATP expression in GBM could be attributed to specific cell-types by using a single-cell analysis pipeline. This segmented IBA1^+^ myeloid cells and UAE-1-lectin^+^ blood vessels to measure the average intensity of OATP isoforms within each cell-type to determine OATP expression in GBM compared to non-tumor tissue. Given the over-expression of all four OATP isoforms in GBM tumor tissue (Figure 1), we studied the immunoreactivity of these markers within defined vasculature and myeloid populations. As aforementioned, OATP1A2, 2B1, 1C1 and 4A1 immunoreactivity was identified on both myeloid cells (IBA1^+^ cells) and blood vessels (Lectin^+^) within both GBM tumor tissue and non-tumor tissue (Figure 3E–H). Hence, we investigated the single-cell expression of each OATP within IBA1^+^ and lectin^+^ masks. Whilst the majority of OATP immunoreactivity was likely attributed to tumor cell expression in GBM tissue (Figure 2), we did not pursue the analysis of the single-cell expression of OATPs on tumor cells in GBM tissue due to the lack of pan-tumor cell markers in addition to the absence of a meaningful comparison to non-tumor cells.

To begin, we compared the tissue-wide average intensity of each OATP to their respective OATP^+^ IBA1^+^ and lectin^+^ blood vessel average intensities. The average intensity of total OATP expression was substantially higher than the average intensity within IBA1^+^ (OATP1A2; 25-fold, OATP2B1; 15-fold, OATP1C1; 32-fold, OATP4A1; 14-fold) masks, with this same trend followed for all four isoforms (Figure 3E–H). A similar trend was also observed when total OATP expression was compared to the average intensity within lectin^+^ OATP1A2; 26-fold, OATP2B1; 15-fold, OATP1C1; 16-fold, OATP4A1; 16-fold) masks. Although OATP^+^ IBA1^+^ cells and OATP^+^ blood vessels only made up a small proportion of the tissue-wide average OATP intensity (Figure 3A–D), we hypothesized that the mean average intensity of each OATP isoform would be higher in GBM vasculature and myeloid populations when compared to their respective non-tumor cells. Overall, single-cell analysis revealed that OATP1A2, 2B1, 1C1 AND 4A1 were indeed expressed on IBA1^+^ cells and lectin^+^ blood vessels. The mean average intensity of OATP1A2 and OATP4A1 was significantly higher in IBA1^+^ cells than in blood vessels (OATP1A2: *P* < .0300, OATP4A1: *P* < .0309) but was relatively similar for OATP2B1 and OATP1C1 within GBM tumor tissue.

Concurrently, we investigated the single-cell expression of OATPs within GBM tumor IBA1^+^ cells and lectin^+^ blood vessels compared to their respective non-tumor cells. Indeed, single-cell analysis revealed that the mean average intensity of OATP1A2 (18-fold, *P* < .0001), OATP2B1 (3-fold, *P* = .0002), OATP1C1 (8-fold, *P* < .0001) and OATP4A1 (5-fold, *P* = .0001) in GBM IBA1^+^ cells was significantly higher than in non-tumor IBA1^+^ cells (Figure 3I–L). The mean single-cell average intensity of these markers reflects the average expression of these proteins within IBA1^+^ populations. Furthermore, the mean average intensity of OATP1A2 (6-fold, *P* < .0001), OATP2B1 (6-fold, *P* < .0001), OATP1C1 (7-fold, *P* = .0001) and OATP4A1(4-fold, *P* = .0004) in GBM blood vessels was significantly higher than in non-tumor blood vessels (Figure 3M–P). Single-stain images of those represented in Figure 3I–P can be found in Supplementary Figures S6 and S7. Therefore, our single-cell analysis revealed an over-expression of all four isoforms in myeloid cells and blood vessels in GBM tissue, suggesting that myeloid cells and vascular cells have the capacity to upregulate these markers in the context of GBM.

### OATP4A1 Expression Correlates with Hypoxia-Related Genes in Peri-Necrotic Regions of GBM Tissue

**Figure 4.**
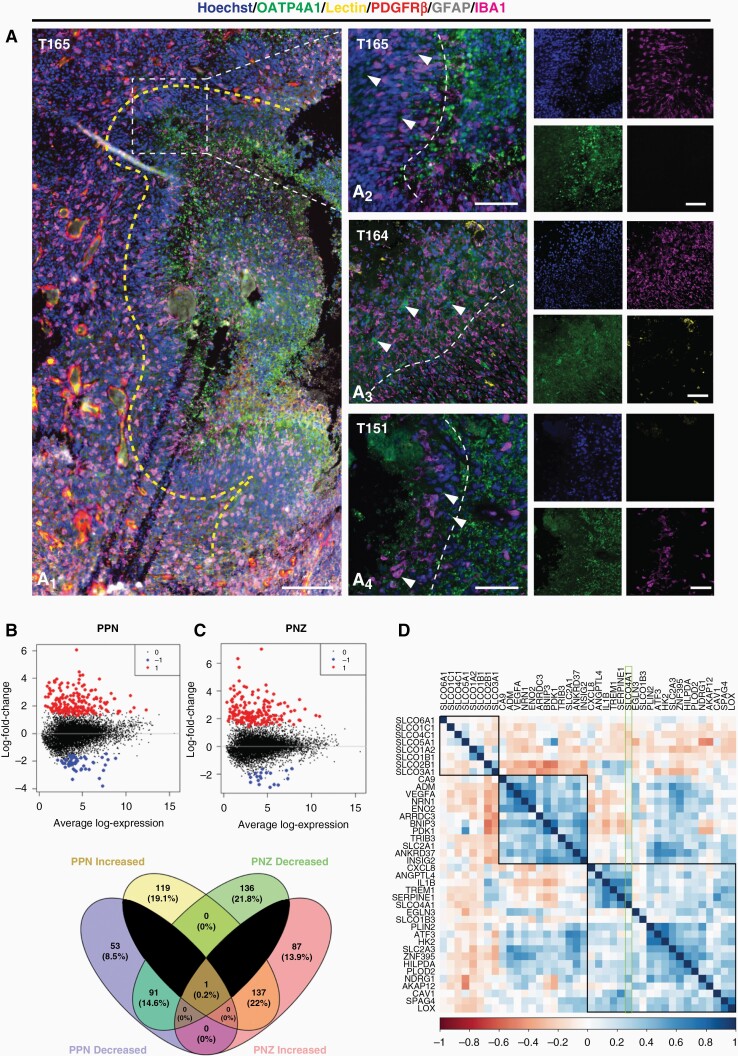
OATP4A1 expression correlate with hypoxia-related genes in PPN and PNZ regions in human GBM tissue. Representative immunohistochemical labelling of OATP4A1 labelled with pan myeloid marker IBA1, tumour cell/astrocyte marker, GFAP, endothelial cell marker, UAE-1 lectin, stromal cell marker, PDGFRβ and Hoechst nuclear counterstain revealed increased expression of OATP4A1 within the PPN microenvironment (A_1_); scale bar = 200 μm. The yellow dotted line delineates the core tumour (left) from the PPN region (right). Immunofluorescent staining of OATP4A1 in PPN regions in three GBM tumour tissue cases (A_2_–A_4_). The white dotted line delineates PPN from PNZ regions. Images taken with a 20x objective; scale bar = 100 μm. Differential gene expression of PNZ (B) and PPN (C) compared to core tumour cells from the Ivy Atlas RNAseq dataset revealed a group of hypoxia-related genes, which were similar between PPN and PNZ (E). Spearman correlation matrix of OATPs and the top 40 DEGs in the PPN and PNZ regions reveal a positive correlation with only the OATP4A1 isoform with hypoxia-related genes (D) .

In contrast to OATP1A2, 2B1 and 1C1, where the immunohistochemical expression was evenly distributed throughout the tumor parenchyma, the expression of OATP4A1 appeared more localized (Figure 4A). Further investigation identified pockets of high OATP4A1 expression in regions adjacent to the PPN in GBM tumor tissue (Figure 4). PPN regions were identified by a lack of lectin-coverage, often adjacent to clear acellular regions, where a hypercellular wave of tumor cells and IBA1^+^ myeloid cells were observed (Figure 4A_1_). Interestingly, qualitative observations found the presence of OATP4A1 staining within the hyper-cellular wave of IBA1^+^ and GFAP^+^ cells; however, this immunoreactivity was greatest directly adjacent to these regions, and reduced towards the periphery (Figure 4 A_2_–A_4_). Given the expression within peri-necrotic regions, we hypothesize that OATP4A1 expression may correlate with hypoxia-related genes in GBM tumor tissue.

Following this observation, we used the publicly available Ivy Atlas dataset to investigate whether OATP4A1 expression correlated with hypoxia-related genes within necrosis niches of the GBM tumor microenvironment (Figure 4). The database examined the gene expression profiles of 42 GBM tumors donated following surgical resection in the context of key anatomical features through laser microdissection for RNAseq.^33^ We specifically investigated differentially expressed genes between the tumor core and the PNZ and PPN regions using a Spearman correlation matrix of gene expression (Figure 4B–C), which revealed a distinct cluster of core tumor-associated, and hypoxia-associated genes (Supplementary Figure S8). Venn diagram analysis of the DEGs revealed 22% of these genes were commonly increased in the PNZ and PPN regions, with an additional 14% of genes reduced in both of these regions (Figure 4D). The top 30 differentially expressed genes in each of these regions were correlated with each of the OATP isoform genes to establish whether OATP were upregulated within these regions (Figure 4D). A Spearman correlation matrix revealed three distinct clusters; a group of eight OATP isoforms, a group of genes, including *CA9, VEGFA, BNIP3* and *PDK1*, commonly associated with hypoxia, and an immunogenic and stress cluster of genes, including *CXCL8, IL1R, TREM1, NDRG1* and *ATF3* (Figure 4D, Supplementary Figure S8). Interestingly, only two OATP isoforms correlated with the expression of these hypoxia-associated genes—*SLCO4A1*. Notably, there was a strong positive correlation of *SLCO4A1* with key hypoxia-induced immunogenic genes *CXCL8, IL1R, TREM1.* In addition, *SLCO4A1* had a strong positive correlation with stress-related hypoxia genes, *ANGPTL4, EGNL3* and *NDRG1* (Figure 4D, Supplementary Figure S8). This observation corroborated our findings *in vitro*, where both the gene and protein expression of OATP4A1 were increased to the greatest extent, compared to OATP1A2, 2B1 and 1C1, in response to hypoxia (Supplementary Figure S5). Indeed, these gene expression profiles corroborated the increased expression of OATP4A1 we observed in the peri-necrotic niche, suggesting OATP4A1 expression may be driven, in part, by hypoxic niches.

## Discussion

This study provides a protein-based validation of OATP gene expression in human GBM tissue using cell type specific markers. Using immunohistochemical quantification methods, we show that OATPs; OATP1A2, 2B1, 1C1 and 4A1 were significantly over-expressed in GBM tissue. Moreover, single-cell analysis revealed that in addition to expression by tumor cells, OATPs were over-expressed on myeloid cells and tumor vasculature. Intriguingly, OATP4A1 expression correlated with hypoxia-related genes and appeared upregulated in PPN regions within human GBM tissue. These findings suggest that tumor and non-tumor cells have the capacity to upregulate OATPs in GBM, and that their expression may vary across the tumor microenvironment. Our findings suggest that the expression of OATPs on both tumor cells and the vasculature may serve as both a potential target for inhibition or to deliver anti-cancer agents to GBM tissue.

One previous study identified protein expression of OATP1A2 and OATP2B1 on human GBM CD31^+^ vasculature; however, they were unable to detect expression on tumor cells.^21^ Yet, in this study, all four OATPs investigated were detected at high levels throughout the tumor parenchyma, and the global expression of these proteins were significantly higher in GBM tissue compared to non-tumor tissue. This corroborates the general trend observed in peripheral malignancies, where OATP expression appears to be specifically upregulated in a number of cancers (Supplementary Table S1). Whilst OATP expression in the normal human brain is low,^34,35^ previous reports suggest that OATPs are expressed on astrocytes with a key role in thyroxine transport.^13,34,36^ Therefore, given the glial origin of GBM, we hypothesized that OATPs would be expressed in GBM tumor cells. Indeed, we found that the most striking OATP expression occurred within the bulk tumor parenchyma, this was reinforced by a positive correlation of all OATPs with GFAP immunoreactivity in GBM tissue, but not in non-tumor brain tissue. However, given the density of tumor cells within GBM tissue, and the lack of a clear tumor cell marker, we were unable to delineate individual tumor cells for single-cell analysis of OATP expression. Future studies utilizing robust tumor-specific markers in human GBM tissue may allow for a more granular analysis of tumor cell OATP expression.

Given the inherent heterogeneity observed within GBM tumors, OATP expression was seen to vary not only between cases, but within the same case. Of particular interest was the expression of OATP4A1 within peri-necrotic regions of the tumor microenvironment. Interestingly, a previous study suggested a possible link between hypoxia and the expression of OATP1B3 in cancer cells,^37^ and others found an upregulation of *SLCO4A1* gene expression in hypoxia in neuroblastoma patients.^38,39^ Despite these findings, an understanding of the tissue-specific context of this signaling axis, particularly in GBM, is lacking. We and others have previously demonstrated *in vitro* that pharmacological stabilization of HIF1-alpha increased the uptake of drugs thought to be transported by OATPs,^7,22,40^ as well as the transcription of OATP genes in patient-derived GBM cell lines (Supplementary Figure S5B). However, DEG analysis utilizing the Ivy Atlas dataset revealed that only *SLCO4A1* positively correlated with hypoxia-related genes within PNZ and PPN regions in GBM patient samples (Figure 4D). This corroborated our immunohistochemical observations of localized expression of OATP4A1 within PPN regions in GBM tissue (Figure 4A), in addition to our *in vitro* findings, where the gene and protein expression of OATP4A1 was regulated in response to hypoxia (Supplementary Figure S5). We found that all four OATPs investigated were expressed across a range of patient-derived GBM cells (Supplementary Figure S5A), and that their expression was regulated by pharmacological stabilization of HIF1-alpha and exposure to physiological hypoxia (Supplementary Figure S5B–G). The present study demonstrates the expression and regulation of OATPs in glioblastoma both *in situ* and *in vitro* and provides the foundations to further explore the functional significance of these transporters in GBM tumorigenesis. We hypothesize that given the hypoxia-dependent regulation of OATP expression, identified both *in situ* and *in vitro*, these transporters may have a putative role in the sodium-independent uptake and surveillance of nutrients in the surrounding microenvironment. Besides, tumor-enhanced expression of several transporter proteins for glucose and amino acids have been well documented, implicating nutrient signaling in tumor cell growth and survival.^41–44^ Therefore, future studies should aim to elucidate the functional significance of these transporters in GBM pathogenesis, and investigate the potential clinical implications of utilizing tumor microenvironment-driven OATP expression for drug targeting.

Previous studies have reported the expression of OATP2 in human microglia, macrophages, monocytes and dendritic cell populations.^45,46^ Given that high myeloid load correlates with poor patient prognosis in a range of solid tumors, including GBM,^26^ we investigated the expression of the OATP isoforms in the IBA1^+^ GBM myeloid populations. Using a single-cell analysis approach, we identified a significant over-expression of all four OATP isoforms by the myeloid cells within GBM tumor tissue, with the proportion of total IBA1^+^ cells positive for OATP immunoreactivity being greater than 90% (Supplementary Figure S9). Interestingly, only OATP2B1 was clearly detected on IBA1^+^ cells in non-tumor tissue (Figure 3G). Targeting the myeloid population in GBM, however, is complicated by the presence of both resident microglia and infiltrating tumor-associated macrophages, which may have diverging roles in GBM.^26^ Therefore, it is imperative that future studies associate the OATP isoforms to each myeloid population using microglia-specific markers, such as P2RY12 and TMEM119.^26^

Furthermore, we revealed significant over-expression of all four OATPs in lectin^+^ blood vessels in GBM tissue. This was consistent with a previous report of OATP1A2 and OATP2B1 expression on CD31^+^ GBM vasculature.^21^ In contrast, OATP expression within the vasculature of non-tumor tissue was scarce, with only weak detection of OATP2B1 and 4A1. Whilst it is generally accepted that OATPs play an important role in the disposition of substrates in cancer therapy, the role of OATPs in BBB transport of cancer therapies is unclear. Critically, the low expression of OATPs we observed in human non-tumor vasculature may contribute to the conflicting evidence on the use of OATPs to improve CNS delivery of therapeutics, where most studies have focused on animal models which have higher brain and vascular expression of OATPs.^47–50^ However, the GBM vascular expression of OATPs supports the hypothesis that OATPs could be an ideal target for the delivery of anti-cancer agents through the GBM BTB. It should be noted that the tissue expression of these isoforms is not synonymous with functionality; in particular, the variable expression of OATP1 family members and their genetic variations have been extensively investigated as a possible source of altered pharmacokinetics and pharmacodynamics of anti-cancer drugs. Therefore, a functional understanding of OATPs and genetic variations, if any, in human tumor vasculature would be prudent for their exploitation as a drug-delivery target. In addition, a characterization of the sub-cellular localization, as in the abluminal and luminal expression profiles of OATPs on endothelial cells, would be paramount to understanding their potential as a drug-delivery target.

## Conclusion

For the first time, we demonstrate that OATP1A2, 2B1, 1C1 and 4A1 proteins are indeed over-expressed throughout the tumor parenchyma in GBM tissue. While OATP expression is highest on GBM tumor cells, OATP isoforms were also highly upregulated by myeloid cells and vasculature within the tumor microenvironment. Similarly, we provide evidence that OATP4A1 was preferentially expressed in the hypoxic peri-necrotic niche, suggesting that local microenvironments can drive specific OATP isoform expression. In summary, this study sheds light on the exciting prospects of elucidating the function of OATPs in GBM pathogenesis and exploiting their over-expression in GBM vasculature and cells to deliver targeted anti-cancer therapeutics.

## Supplementary Material

vdac166_suppl_Supplementary_Material
