# Supplementary material for: Single-cell image analysis reveals over-expression of organic anion transporting polypeptides (OATPs) in human glioblastoma tissue
Source: Neurooncol Adv. 2022 Oct 14;4(1):vdac166. doi: 10.1093/noajnl/vdac166 (PMC9653174; doi:10.1093/noajnl/vdac166)
Supplement: vdac166_suppl_Supplementary_Material [file vdac166_suppl_supplementary_material.docx]

**Supplementary Table S1 Expression of OATPs in malignant and non-malignant tissue**

| **OATP Isoform** | **Non-malignant Tissue** | **Malignant Tissue** | |
| --- | --- | --- | --- |
|  |  | Increased Expression | Decreased Expression |
| OATP1A2 | Blood brain barrier^1,2^  Enterocytes^2^  Cholangiocytes^2^  Kidneys^3^ | Breast cancer^4^  Prostate cancer^5^  Bone cancer^6^ | Colon Cancer^7^ |
| OATP1B1 | Liver ^8^ | Colon Cancer^7^  Ovarian Cancer^9^ | Liver Cancer^10,11^ |
| OATP1B3 | Liver^12,13^ | Colon cancer^7^  Pancreatic cancer^14,15^  Lung cancer^16^  Prostate cancer^17,18^  Breast Cancer^19^  Testicular Cancer 85  Ovarian Cancer^9^ | Liver Cancer^20^ |
| OATP1C1 | Brain^15^  Testis^15^  Ciliary body epithelium^21^ | Bone Cancer^6^ |  |
| OATP2A1 | Ubiquitous^22^ | Breast Cancer^23^  Liver Cancer^24^  Bone Cancer^6^ | Bowel Cancer^25^  Stomach Cancer^25^  Ovarian Cancer^9,25^  Lung Cancer^25^  Kidney Cancer^25^ |
| OATP2B1 | Blood brain barrier^26^  Heart^27^  Enterocytes^28^  Placenta^29^ | Bone Cancer^6^  Breast Cancer^23^  Prostate Cancer^172^ | Liver Cancer^30^  Pancreatic Cancer^30^ |
| OATP3A1 | Ubiquitous | Bone Cancer^6^  Breast Cancer^23^  Liver Cancer |  |
| OATP4A1 | Ubiquitous | Breast Cancer^23^  Liver Cancer  Colon Cancer^7^ |  |
| OATP4C1 | Kidneys | Breast Cancer^23^ |  |

Supplementary Table S2 Paraffin-embedded tissue was used from GBM tumour and epilepsy (non-tumour) middle temporal gyrus to characterise organic-anion-transporting polypeptides.

| **Case number** | **Age** | **Sex** | **Pathology** | **MGMT status** |
| --- | --- | --- | --- | --- |
| E204 | 45 | F | Epilepsy | N/A |
| E211 | 51 | F | Epilepsy | N/A |
| E201 | 26 | M | Epilepsy | N/A |
| E214 | 35 | F | Epilepsy | N/A |
| E215 | 29 | F | Epilepsy | N/A |
| E217 | 30 | M | Epilepsy | N/A |
| E213 | 34 | M | Epilepsy | N/A |
| E212 | 40 | F | Epilepsy | N/A |
| T017 | 57 | M | GBM | Unmethylated |
| T019 | 64 | M | GBM | Unmethylated |
| T039 | 68 | F | GBM | Unmethylated |
| T072 | 57 | F | GBM | Methylated |
| T084 | 69 | M | GBM | Methylated |
| T090 | 54 | F | GBM | Methylated |
| T100 | 43 | M | GBM | Unmethylated |
| T102 | 52 | M | GBM | Unmethylated |
| T104 | 63 | M | GBM | Unmethylated |
| T107 | 75 | M | GBM | Methylated |
| T113 | 58 | M | GBM | Methylated |
| T115 | 67 | M | GBM | Unmethylated |
| T116 | 54 | M | GBM | Unmethylated |
| T119 | 80 | M | GBM | Unmethylated |
| T120 | 75 | F | GBM | Unmethylated |
| T124 | 62 | M | GBM | Unmethylated |
| T135 | 54 | F | GBM | Unknown |
| T141 | 61 | M | GBM | Unmethylated |
| T146 | 60 | M | GBM | Methylated |
| T147 | 47 | F | GBM (mut) | Unmethylated |
| T151 | 65 | M | GBM | Methylated |
| T152 | 66 | F | GBM | Methylated |
| T164 | 53 | F | GBM | Unmethylated |
| T165 | 47 | M | GBM | Unmethylated |

Supplementary Table S3 Non-tumour (epilepsy) and glioblastoma tumour human cases was used for the *in situ* and *in vitro* characterisation of the gene expression of organic-anion transporting polypeptides by RT-qPCR

| **Case number** | **Age** | **Sex** | **Pathology** | **MGMT Status** |
| --- | --- | --- | --- | --- |
| E202 | 32 | F | Epilepsy | N/A |
| E205 | 31 | M | Epilepsy | N/A |
| E215 | 29 | F | Epilepsy | N/A |
| T069 | 70 | M | GBM | Methylated |
| T073 | 55 | F | GBM | Unmethylated |
| T084 | 69 | M | GBM | Methylated |
| T115 | 67 | M | GBM | Unmethylated |
| T146 | 60 | M | GBM | Methylated |
| T141 | 61 | M | GBM | Unmethylated |

**Supplementary Table S4 List of primers used for qRT-PCR**

| **Accession** | **Gene (protein)** | **Sequence (5’ to 3’)** | | **Amplicon (bp)** |
| --- | --- | --- | --- | --- |
| NM_001101 | *ACTB* | Fw | TGGTGGGCATGGGTCAGAAGGA | 94 |
|  | (β-actin) | Rv | ATGCCGTGCTCGATGGGGTACT |  |
| NM_007256 | *SLCO2B1* | Fw | TCTCCGGCTACCTAAAGAGCT | 100 |
|  | (OATP2B1) | Rv | TTCCCCACCTCGTTGAAGGA |  |
| NM_147216.2 | *SLCO1C1* | Fw | AACTCCCATTCAGCCTTTGG | 81 |
|  | (OATP1C1) | Rv | CCGTCTGCACACACCCAATA |  |
| NM_005075 | *SLCO1A2* | Fw | CGCAGGATCCATCAGAGTGTA | 92 |
|  | (OATP1A2) | Rv | AGTTTCACCCATTCCACGTACA |  |
| NM_016354 | *SLCO4A1* | Fw | CTGTACAAGGTGCTGGGCGT | 79 |
|  | (OATP4A1) | Rv | GGCCATCTGAAGACTCCGAC |  |


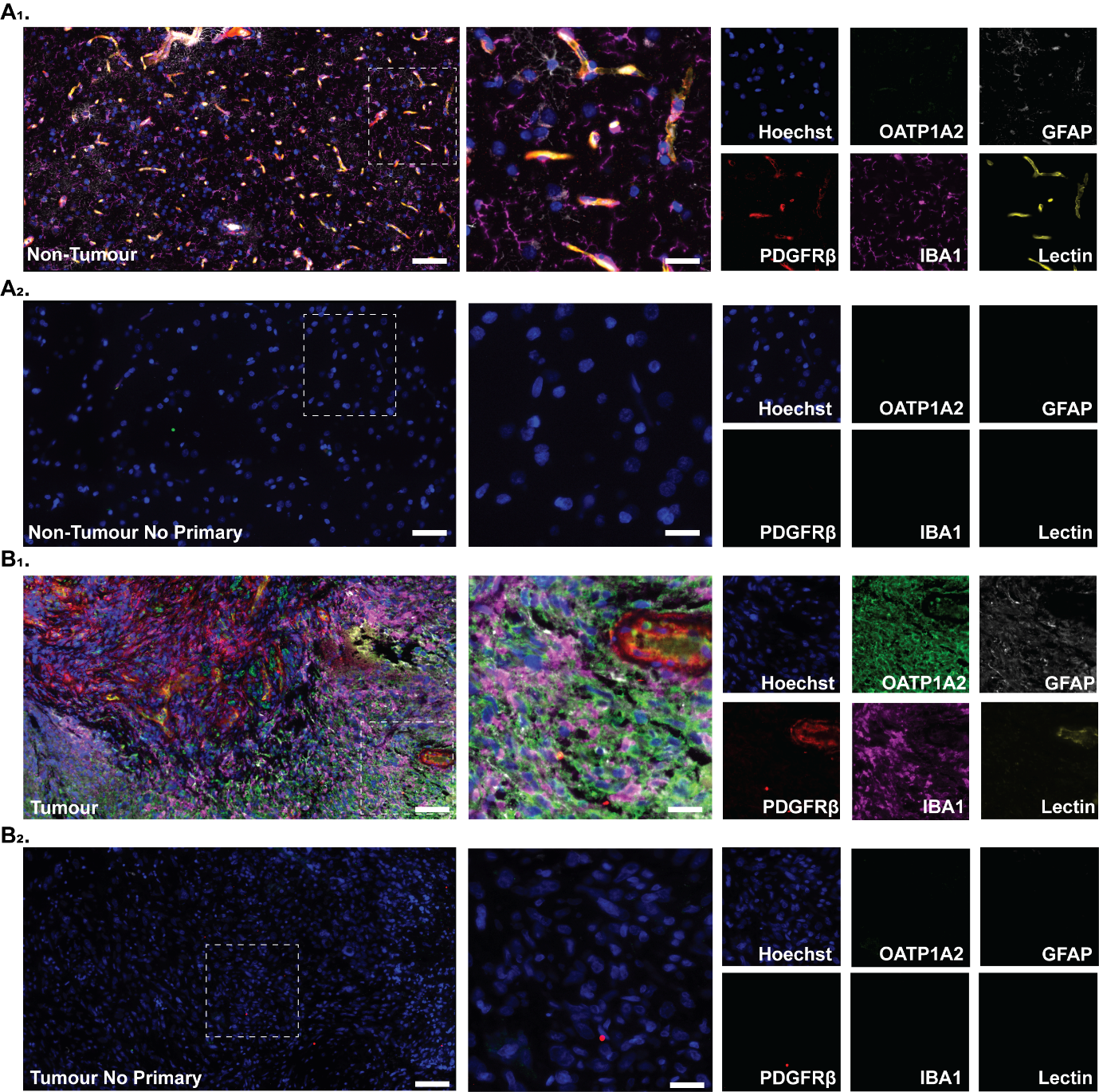


Supplementary Figure S1 No primary controls of immunohistochemical staining in tumour and non-tumour tissue. Representative immunohistochemical labelling of OATP1A2, as labelled (green) with GFAP, stromal cell marker PDGFRβ (red), pan myeloid marker, IBA1 (magenta), endothelial cell marker UAE-lectin (yellow); and Hoechst nuclear counterstain (blue) in non-tumour and GBM tissue, scale bar = 20 μm.

First, we sought to quantify the tissue-wide expression of each OATP, as well as pan-tumour cell marker, GFAP, pan myeloid marker, IBA1, endothelial marker, UAE-1-lectin, and stromal cell marker, PDGFRβ. Binary masks of positive immunoreactivity for each marker were created using the threshold clip tool. Using these binary masks, the area coverage and the integrated intensity of each marker were measured. The area coverage was presented as the mean sum staining area of each marker of interest (MOI) normalised to the sum region of interest (ROI), as a percentage. Similarly, the integrated intensity was presented as the mean integrated intensity per case normalised to ROI area. The mean integrated intensity per ROI measurement significantly positively correlated with area coverage measures for each MOI (OATP1A2, r = 0.9008, p < 0.0001; OATP2B1, r = 0.5356, p = 0.0029; OATP1C1, r = 0.6648; OATP4A1, r = 0.8400, p < 0.0001) (Supplementary Figure S4). Each dot represents a single case.

We subsequently sought to quantify the single-cell expression of OATP1A2, 2B1, 1C1, and 4A1 within myeloid cells, blood vessels, and stromal PDGFRβ-positive cells. These analyses were based on previously described single-cell image analysis pipelines ^31,32^. Briefly, binary masks of IBA1, lectin, and PDGFRβ were generated using the adaptive threshold tool. These binary masks were used to score each object, or cell, as a myeloid cell (IBA1-positive), a blood vessel (lectin-positive), or a stromal PDGFRβ-positive cell (PDGFRβ-positive and lectin-negative). The analysis of the expression of each MOI within each cell type is described below, and can be visualised by the workflow in Supplementary Figure S2 and S3.

*Myeloid cells:* The average intensity of each OATP transporter was measured in each IBA1-positive object. The mean average intensity per case was presented.

*Blood vessels:* For complete identification of all blood vessels, the lectin binary mask was first dilated circularly by 15 pixels. Each object within the lectin binary mask was considered a blood vessel, and therefore, to determine OATP expression in blood vessels, the average intensity of each OATP transporter was measured in each lectin-positive object. The mean average intensity of each OATP per vessel was then averaged across all vessels for each section. The mean average intensity per case was presented, where each dot represents a single case.

*Stromal PDGFRβ-positive cells*: Each object within the PDGFRβ binary mask was considered a cell and the average intensity of each OATP transporter was measured in each PDGFRβ-positive object. However, it was unclear whether these PDGFRβ-positive cells were stromal or blood vessel associated. To differentiate stromal PDGFRβ from vascular PDGFRβ, overlay of PDGFRβ-positive cells with lectin-positive blood vessels was assessed. If any part of a PDGFRβ-positive cell overlayed a lectin-positive blood vessel, it was considered ‘blood-vessel associated’. In contrast, if a PDGFRβ-positive cell did not overlay a lectin-positive blood vessel, it was considered ‘stromal’. OATP expression was only assessed in stromal PDGFRβ-positive cells, as OATP expression in blood vessel-associated PDGFRβ-positive cells was encompassed in the blood vessel analysis. Of note, non-vascular associated (stromal) PDGFRβ-positive cells are not found in non-tumour brain tissue, hence comparisons between tumour and non-tumour tissue were not made. All data are presented as mean average intensity per case, where each dot represents a single case.


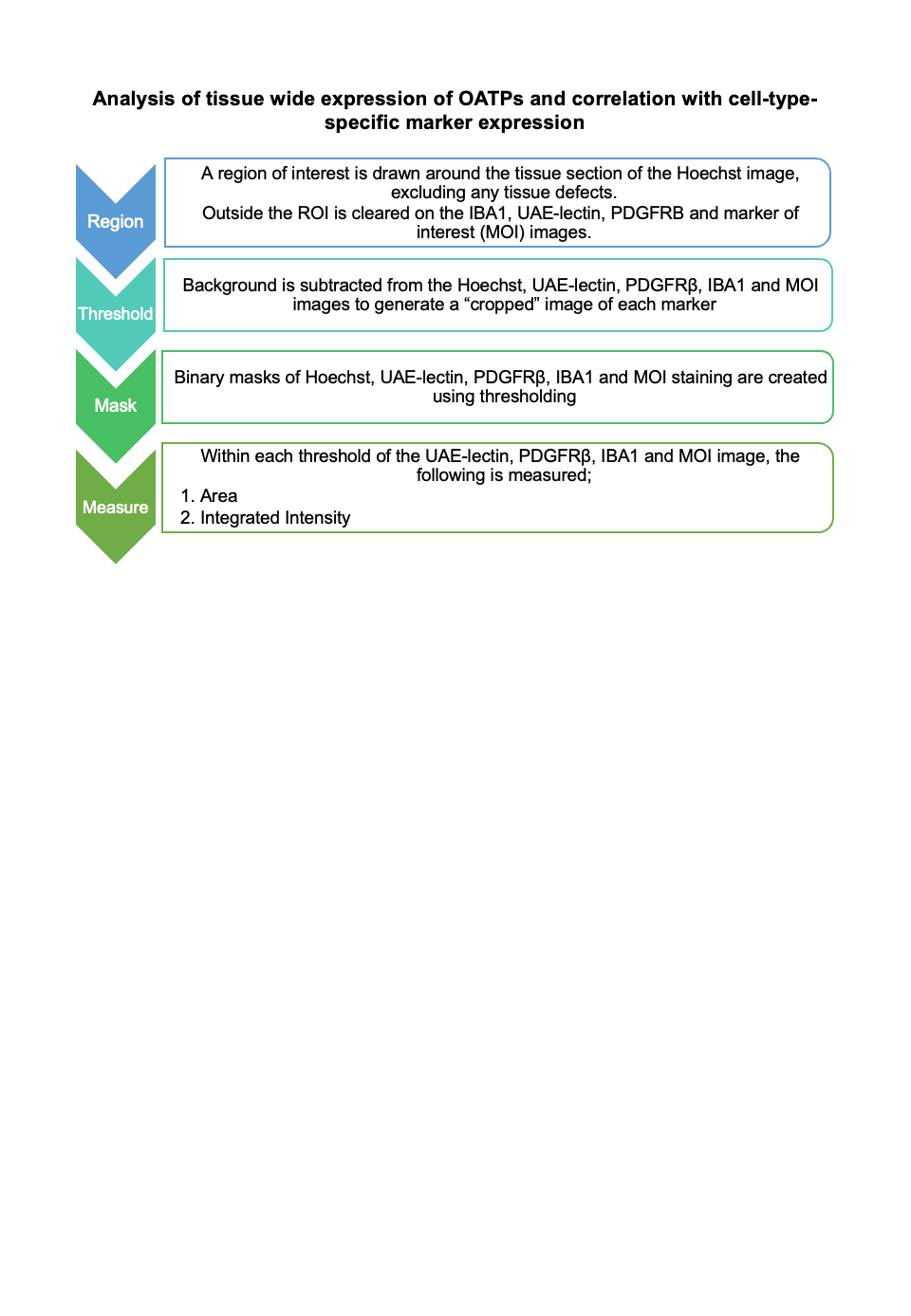


Supplementary Figure S2 Flow diagram summary of the integrated intensity and density analysis of OATP expression in glioblastoma tissue

Abbreviations: MOI: marker of interest, ROI: region of interest, PDGFRβ: platelet-derived growth factor beta.


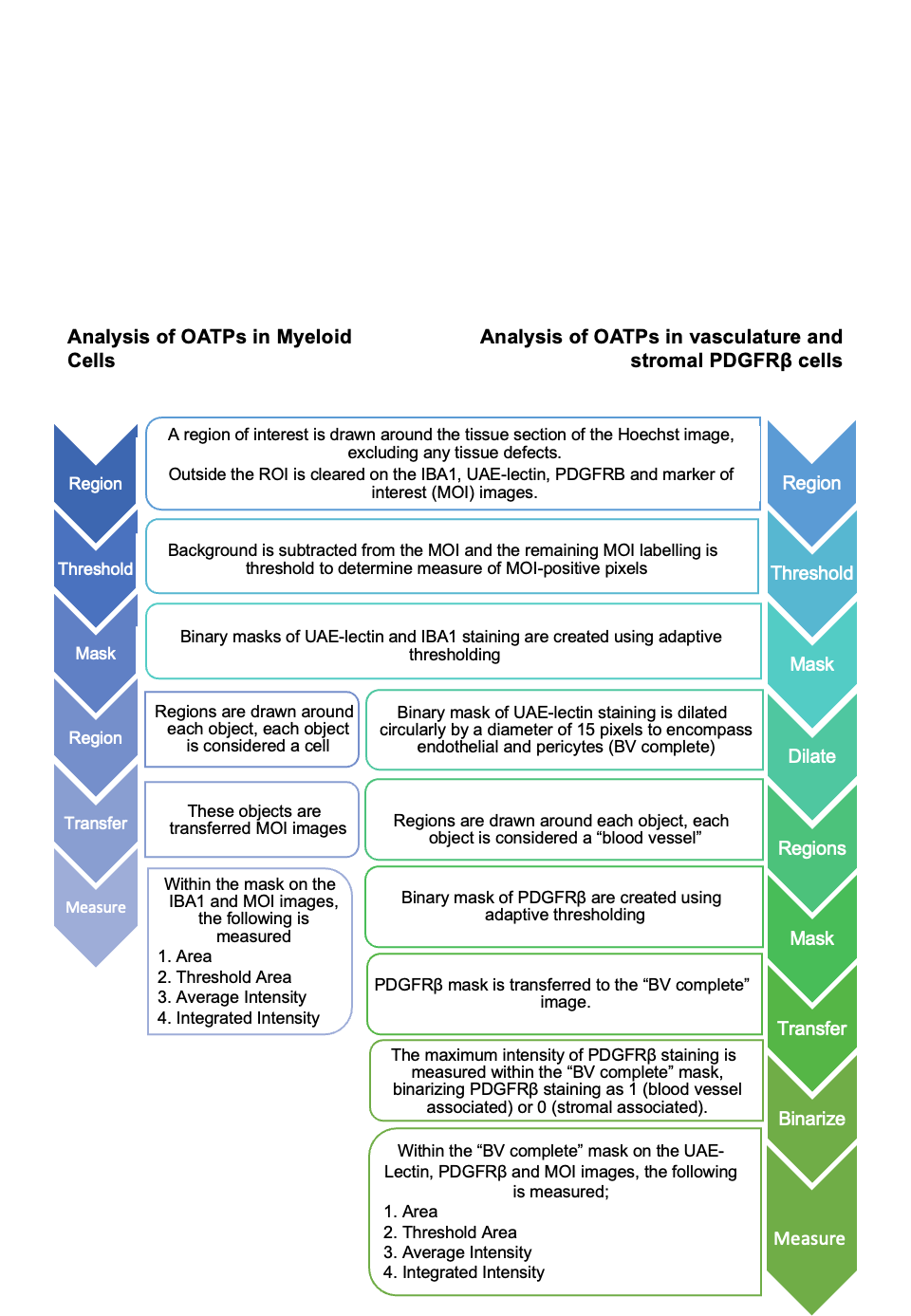


Supplementary Figure S3 Flow diagram summary of the single-cell integrated intensity and density analysis of OATP expression in glioblastoma tissue.

Abbreviations: MOI: marker of interest, ROI: region of interest, PDGFRβ: platelet-derived growth factor beta.

### Biopsy and culture of human glioblastoma brain tissue

GBM tumour samples were obtained from patients undergoing therapeutic tumour resections at the Auckland City Hospital. All specimens were collected with full informed patient consent and ethical approval was obtained from the Health and Disabilities Ethics Committee (New Zealand). All methods were in accordance with the approved guidelines and regulations. The tissues were diced into 1 mm^3^ fragments, followed by 30 min of enzymatic dissociation (10 U/mL DNase (Invitrogen) and 2.5 U/mL papain (Worthington) in Hibernate-A medium (Gibco)) at 37° C. The enzymatic incubation was stopped by the addition of an equal volume of GBM cell culture medium comprised of DMEM/F12 (Gibco), supplemented with 2% B27 minus vitamin A (Gibco), 1% penicillin-streptomycin (PS; Gibco), and 1% GlutaMAX (Gibco). The cell suspension was passed through a 70 μm cell strainer (Becton Dickenson, NJ, USA), centrifuged for 7 min at 170 x g, and seeded into T75 culture flasks (Nunclon™ Delta surface flasks; Nunc) in GBM cell media and incubated overnight at 37 °C with 95% air/5% CO_2_. The following day, the non-adherent tumour cells were collected, centrifuged at 170 x g for 5 min, resuspended in culture medium, and added to a new culture flask. The adherent (myeloid) cells that remained in the original culture flasks were used for other experiments. Often, the GBM cells grew as spheroids. When the spheres reached ~300 µm in diameter, they were dissociated into single cells by Accutase® cell dissociation solution (Invitrogen) and either passaged or plated for experimentation. A summary of the pathological information from Auckland City Hospital of the patient cases used in this study is seen in Supplementary Table S3.

## Immunocytochemical staining of patient-derived GBM cells

For *in vitro* characterisation, patient-derived GBM cells (n = 4 cases) were seeded on 96-well plates for immunocytochemistry, and 6-well plates for RNA extraction (Matrigel® coated, for GBM cells, 50,000 cells/mL) for 72 hours. A half-medium change was performed before cells were exposed to hypoxia for 4 hours. At end-point, an acute plate was fixed after 4 hours exposure to hypoxia to validate hypoxia changes with carbonic-anhydrase IX, HIF1-alpha and p53, and RNA was extracted from the 6-well plates. In addition, a second 96-well plate was transferred to normoxia for 24 hours to investigate protein changes in OATP expression.

At endpoint, glioblastoma cells were fixed using 4 % paraformaldehyde solution. After cells were washed and permeabilized in PBS with 0.2 % Triton X-100™ (PBS-T), they were incubated with primary antibodies overnight at 4 °C (all antibodies were diluted in goat immunobuffer (1 % goat serum, 0.2 % Triton X-100™, and 0.04 % thiomersal in PBS)). Dilutions and sources of antibodies are listed in Supplementary Table S5. Plates were washed again in PBS-T, incubated with secondary antibodies for 2–3 h at room temperature, and then rinsed. Nuclei were detected using Hoechst (33258 Sigma, St. Louise, MO, USA).

Supplementary Table S5. Antibodies and dilutions used in this study

|  | **Company** | **Catalogue number** | **Concentration** |
| --- | --- | --- | --- |
| Primary Antibodies |  |  |  |
| Mouse anti-B-actin | Abcam | Ab6277 | 1:10000 |
| Mouse anti-Carbonic-Anhydrase IX | Thermofisher | MA5-16318 | 1:500 |
| Mouse anti-HIF1-alpha | Abcam | Ab51608 | 1:500 |
| Rabbit anti-OATP1A2 | Abcam | Ab221804 | 1:1000 |
| Rabbit anti-p53 | Abcam | Ab26 | 1:500 |
| Rabbit anti-SLCO2B1 | Thermofisher | PA5-42453 | 1:500 |
| Rabbit anti-SLCO1C1 | Abcam | Ab234729 | 1:500 |
| Rabbit anti-SLCO4A1 | Sigma-Aldrich | HPA030669 | 1:500 |
| Secondary Antibodies |  |  |  |
| Goat anti-rabbit Alexa 647 | Life Technologies | A-21244 | 1:500 |
| Goat anti-mouse Alexa IgG_2_ 488 | Life Technologies | A-21131 | 1:500 |
| Goat anti-mouse Alexa IgG_1_ 594 | Life Technologies | A-21125 | 1:500 |
| Goat anti-rabbit Alexa 594 | Life Technologies | A-11012 | 1:500 |
| Goat anti-mouse Alexa 488 | Life Technologies | A-11001 | 1:500 |

### Acquisition and analysis of immunocytochemistry images

Image acquisition was done using the ImageXpress micro XLS™ (Molecular Devices) high-content screening system, housed at the Biomedical Imaging Research Unit, University of Auckland. Images were acquired from micro-well plates using the ×10/0.3 Plan or ×20/0.45 NA CFI Super Plan Fluor ELWD ADM objective lens and Lumencor Spectra X configurable light engine source. Excitation and emission filters used are listed in Supplementary Table S6.

Supplementary Table S6. Excitation and emission parameters for ImageXpress micro XLS™ high content screening system

| **Cube** | **Filters** | **Lumencor light engine** | **Ex range** | **Ex peak** | **Dichroic** | **Em range** | **Em peak** |
| --- | --- | --- | --- | --- | --- | --- | --- |
| Triple 4 | DAPI | UV (380–410) | 381–399 | 390/18 | 436 | 445–469 | 457 |
| Triple 4 | FITC | Cyan (460–490) | 484–504 | 494/20 | 514 | 518–542 | 530 |
| Triple 4 | TRED | Green (535- 600) | 561–590 | 575/25 | 604 | 612–643 | 628 |
| Quad 5 | DAPI | UV (380–410) | 381–399 | 390/18 | 410 | 419–460 | 440 |
| Quad 5 | FITC | Cyan (460–490) | 474–496 | 485/20 | 504 | 507/533 | 521 |
| Quad 5 | CY5 | Red (620–750) | 644–656 | 650/13 | 669 | 675–723 | 700 |

Microscope information for all image acquisition in this study including light source, filters, excitation, and emission range and peaks in nanometer

## RT-qPCR analysis of the *in vitro and in situ* gene expression profiles of OATPs in GBM tumour and non-tumour cases

For *in vitro* characterisation, patient-derived GBM cells (n = 7 cases) and non-tumour primary patient-derived pericyte cells (n = 6 cases) were seeded on 6-well plates (Matrigel® coated, for GBM cells, 50,000 cells/mL) for 72 hours. A half-medium change was performed before cells were treated with 250 μM pan- OATP inhibitor, sodium taurocholate, and 100 μM 3,4 dihydroxybenzoic acid, a prolyl hydroxylase inhibitor, for 24 hours. Non-tumour patient-derived pericyte cells were left untreated for basal comparisons.

At endpoint, cells were washed in phosphate-buffered saline (PBS) and RNA extraction and purification was performed using the RNeasy® mini kit (Qiagen, Limberg Netherlands) or the RNAqueous®-Micro Total RNA isolation Kit (Ambion, CA, USA) as per manufacturer’s instructions. qRT-PCR was performed using Platinum® SYBR® Green qPCR SuperMix-UDG with Rox (Life Technologies) on a 7900HT Fast Real-Time PCR system as previously described ^33^. Standard curves were run for all primers and efficiencies were all 100 ± 10% . Primer sequences are described in Supplementary Table S4. Relative gene expression analysis was performed normalising the Ct of each primer of interest to the housekeeping gene B-actin.


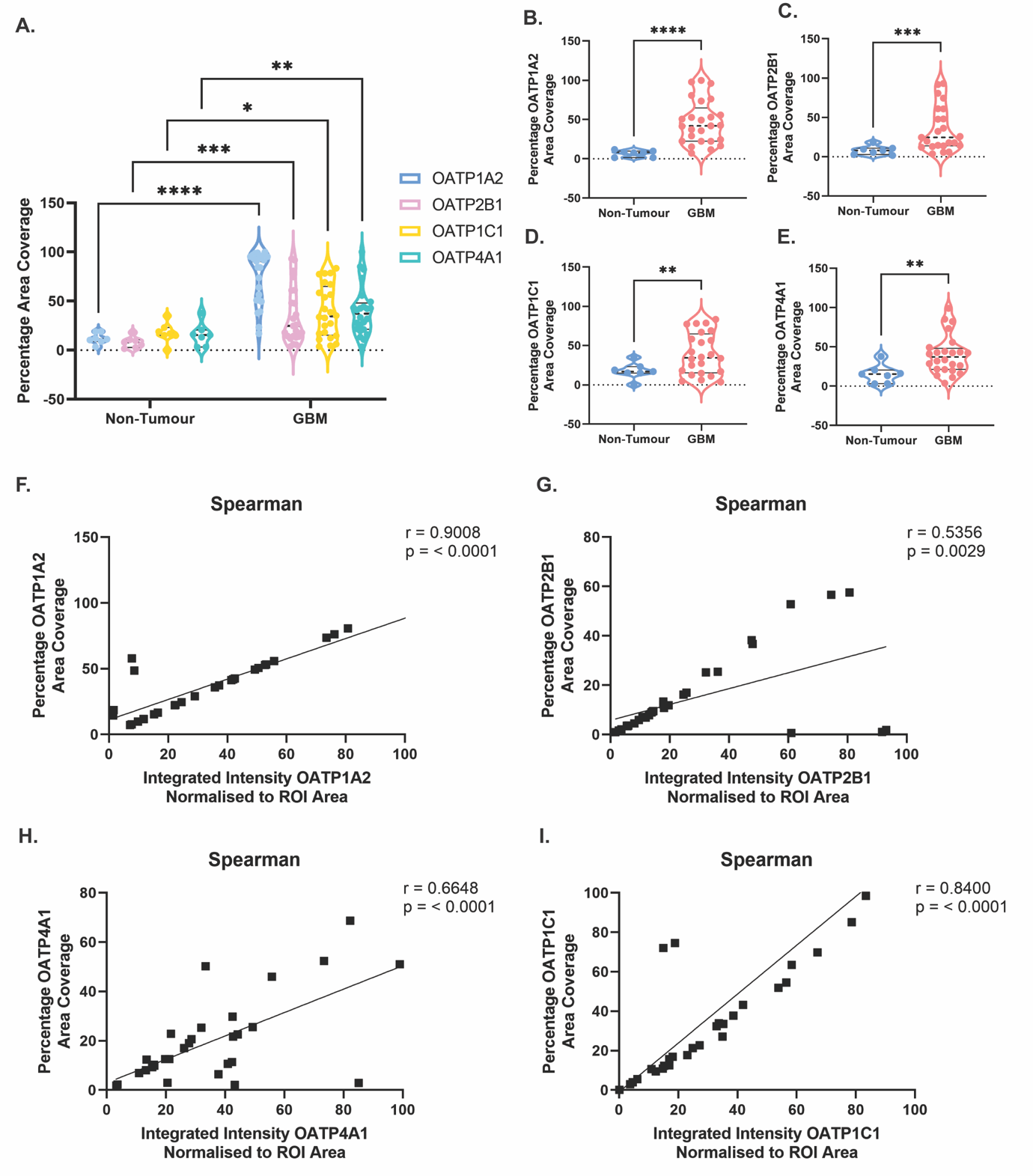


Supplementary Figure S4 Area coverage analysis of the tissue wide expression of OATP1A2, OATP2B1, OATP1C1 and OATP4A1 in glioblastoma and non-tumour tissue. OATP1A2, OATP2B1, OATP1C1 and OATP4A1 was compared between GBM and non-tumour with a one-way ANOVA (A), data presented as violin plot, median ± confidence interval. Each of OATP1A2 (B), OATP2B1 (C), OATP1C1 (D) and OATP4A1 (E) was individually compared between GBM and non-tumour tissue with a Mann Whitney test, data present as median ± confidence interval; * p < 0.05, ** p < 0.01, *** p <0.0001, **** p < 0.00001. Spearman correlation analysis of the relationship between area coverage analysis and integrated intensity analysis, normalised to ROI area, for each OATP isoform (F-I).


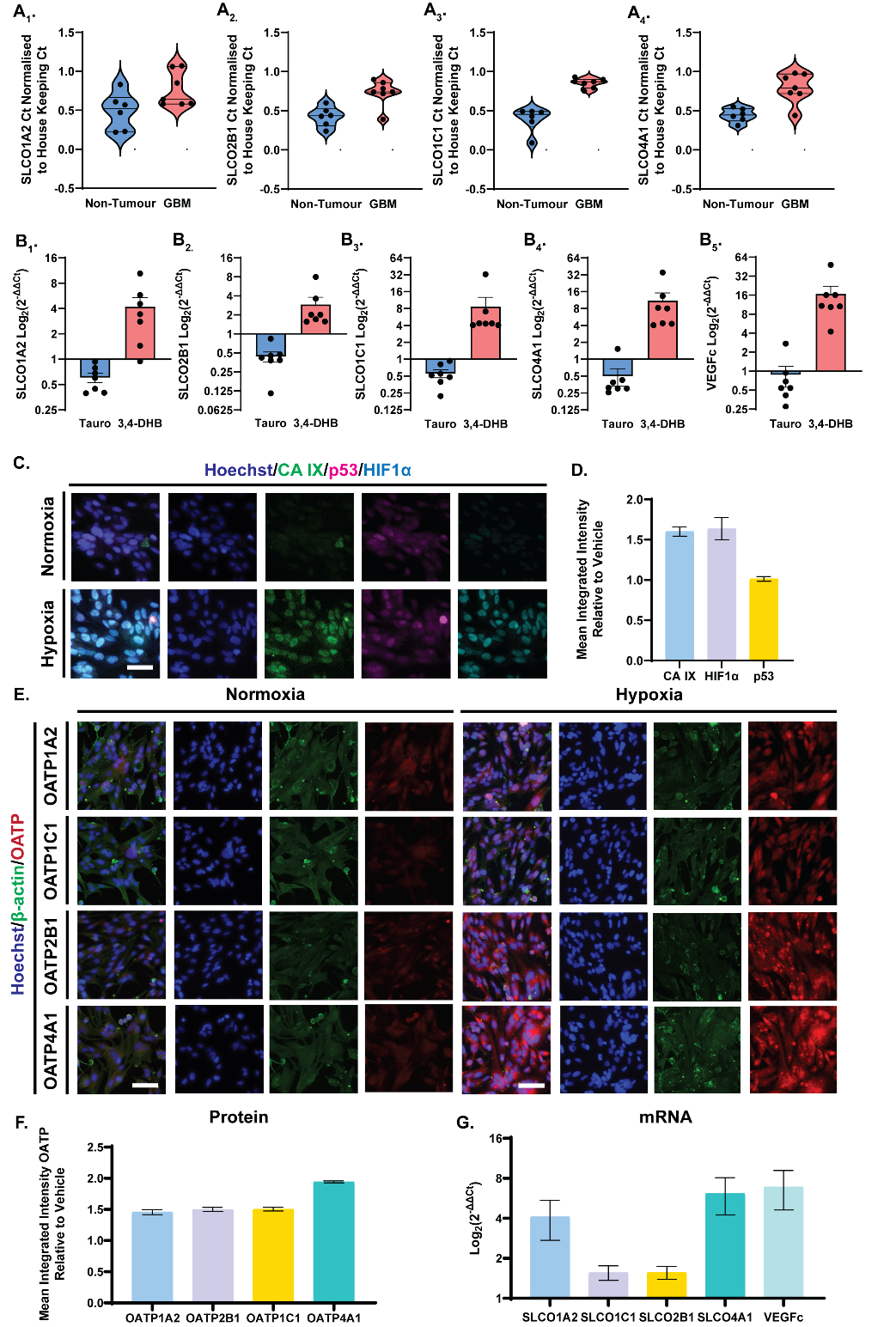


Supplementary Figure S5 mRNA and immunocytochemical expression of OATP1A2, OATP2B1, OATP1C1 and OATP4A1 in patient-derived glioblastoma cells. Basal mRNA levels of each OATP isoform in patient-derived glioblastoma cells compared to non-neoplastic patient-derived pericyte brain cells, represented as GOI Ct normalised to housekeeping Ct, data presented as violin plot, median ± confidence interval (A). OATP isoform expression was increased following treatment with 100 μM 3,4-DHB (24 hours), and treatment with 250 μM pan-OATP inhibitor sodium taurocholate (24 hours) reduced the mRNA levels of OATP isoforms (B). Exposure of GBM cells to hypoxia for 4 hours increased the expression of key hypoxia proteins CA IX, HIF1α and p53 (C-D). Hypoxia also increased the protein expression of all four OATP isoforms (24 hours after returning to normoxia) (E-F) and increased their transcription (4 hours hypoxia) (G). Data represents mean ± SEM for at least four different patient-derived cell lines (B-G).


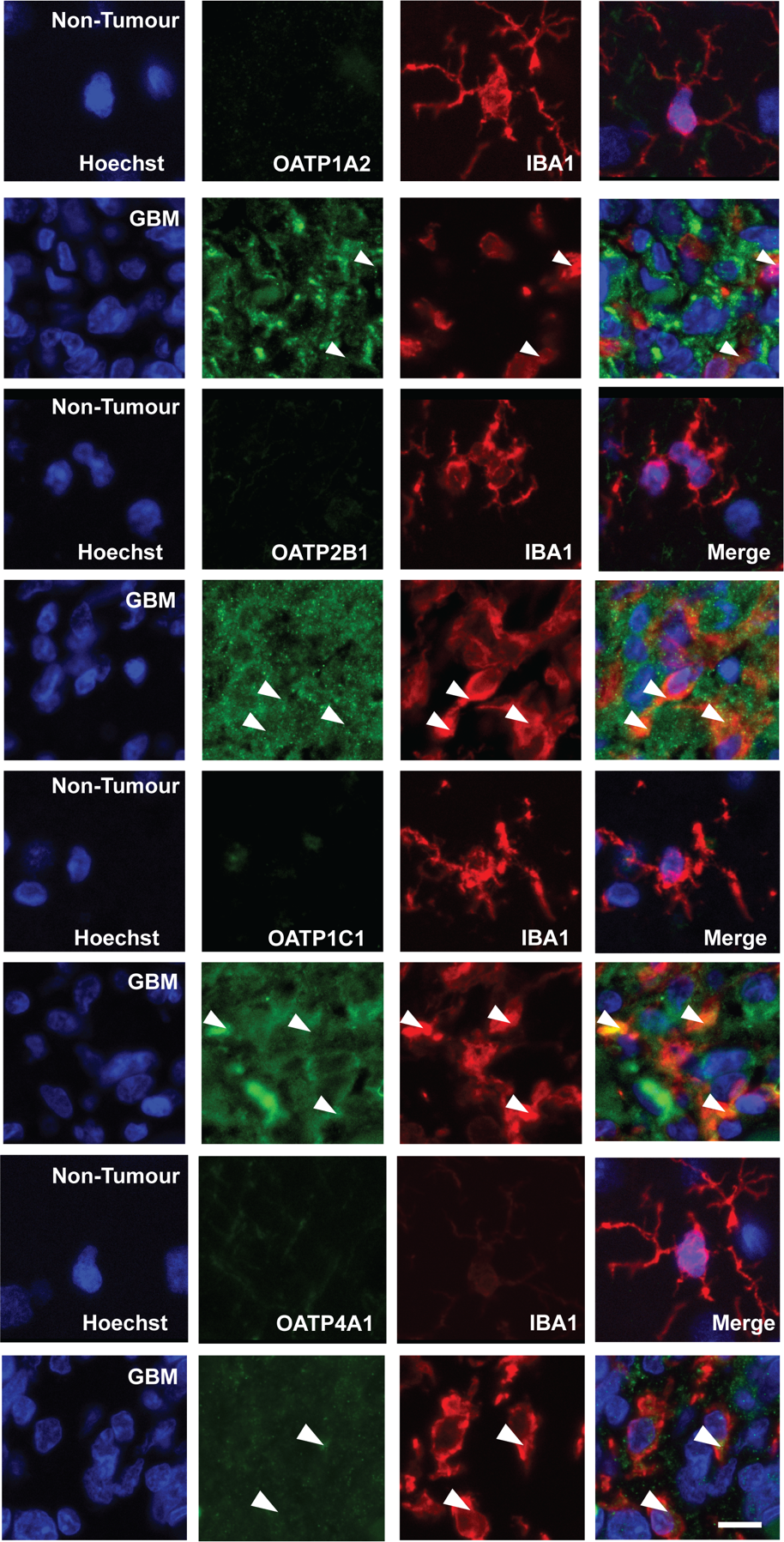


Supplementary Figure S6 Single-stain representative images of OATP isoforms in GBM and non-tumour IBA1-positive cells. Representative immunohistochemical labelling of OATP1A2, 2B1, 1C1 and 4A1, as labelled (green) with pan myeloid marker, IBA1 (red); and Hoechst nuclear counterstain (blue) in non-tumour and GBM tissue scale bar = 10 μm.


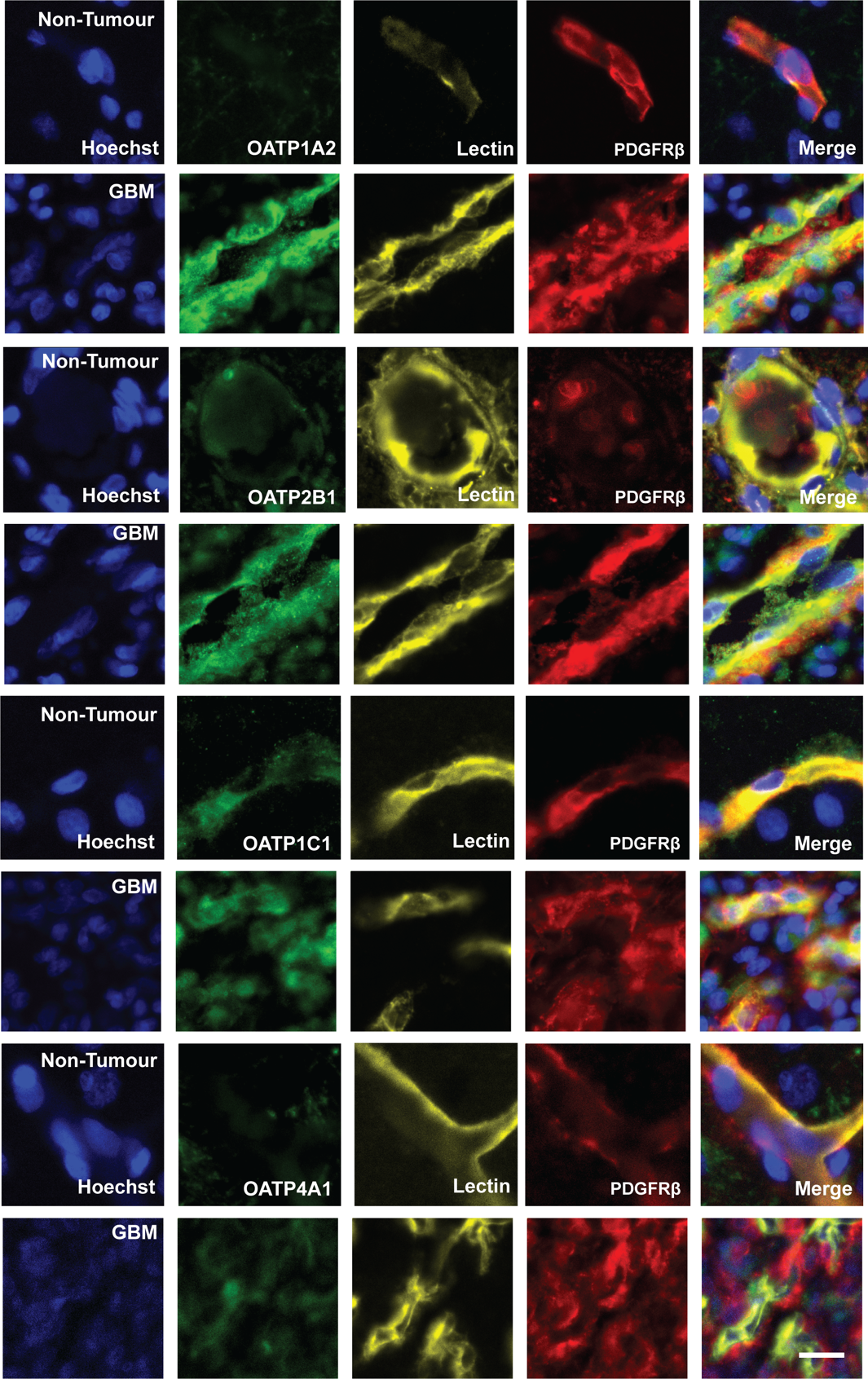


Supplementary Figure S7 Single-stain representative images of OATP isoforms in GBM and non-tumour lectin-positive blood vessels. Representative immunohistochemical labelling of OATP1A2, 2B1, 1C1 and 4A1, as labelled (green) with endothelial cell marker, UAE-1 lectin (yellow); stromal cell marker, PDGFRβ (red); and Hoechst nuclear counterstain (blue) in non-tumour and GBM tissue scale bar = 10 μm.


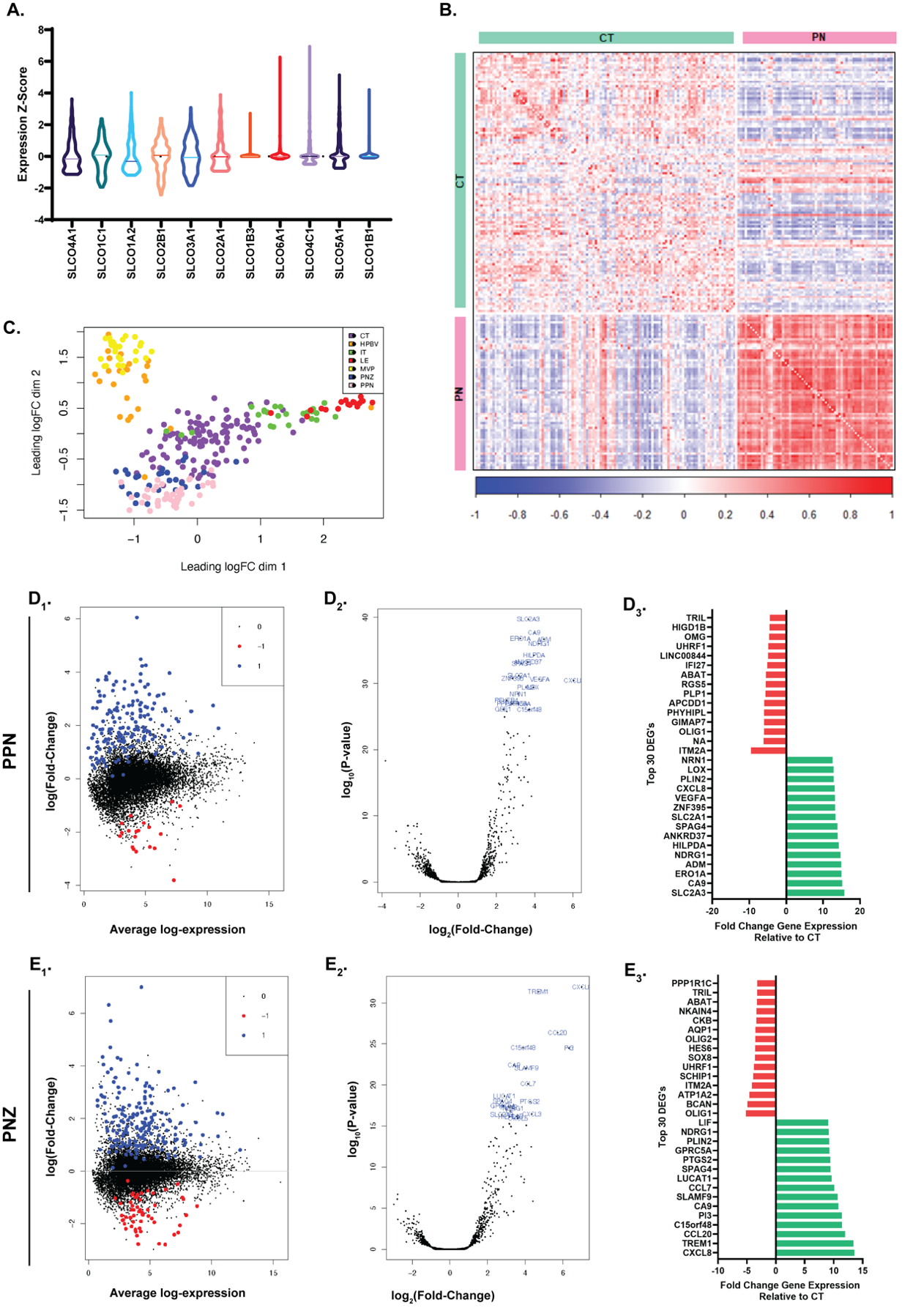


**Supplementary Figure S8 Differential gene expression between perinecrotic and pseudo palisading necrosis regions and the core tumour from the IVY Atlas RNAseq dataset**. IVY Atlas reveals mRNA expression of six of the OATP isoforms in glioblastoma tumour samples. Spearman correlation matrix reveals distinct gene clusters between PNZ and PPN regions core tumour regions (B). MDS plots highlight region-specific clustering of samples. Differential gene expression of PNZ (D) and PPN (E) with core tumour cells from the IVY Atlas RNAseq dataset revealed an upregulation of a group of hypoxia-related genes


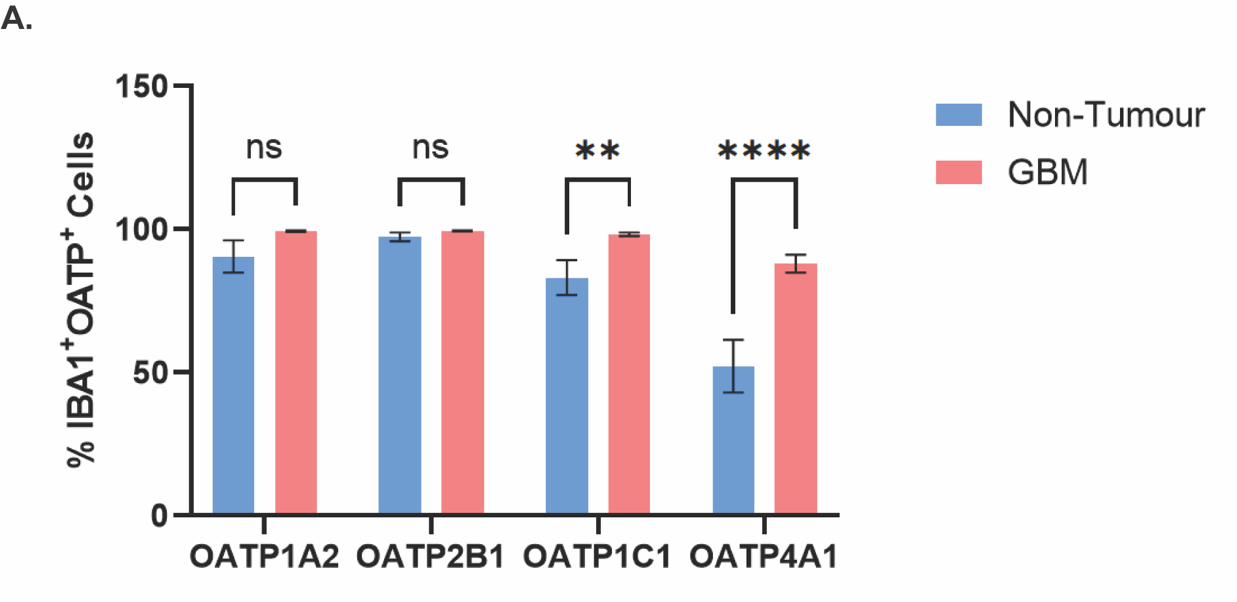


Supplementary Figure S9 Single cell analysis of the percentage of OATP^+^IBA1^+^ Cells in glioblastoma and non-tumour tissue.

Supplementary Table S4 Correlation analysis of integrated intensity of markers of interest with GFAP, IBA1, PDGFRβ, UAE-lectin, and each OATP isoform (OATP1A2, OATP2B1, OATP1C1, OATP4A1 in GBM neurosurgical tissue.

| **Marker of Interest** | **OATP1A2** | | | **OATP2B1** | | | **OATP1C1** | | | **OATP4A1** | | |
| --- | --- | --- | --- | --- | --- | --- | --- | --- | --- | --- | --- | --- |
|  | **r** | **P value** | **Summary** | **r** | **P value** | **Summary** | **r** | **P value** | **Summary** | **r** | **P value** | **Summary** |
| **GFAP** | 0.8584 | < 0.00001 | **** | 0.2443 | 0.2613 | ns | 0.4703 | 0.0235 | * | 0.3597 | 0.0919 | ns |
| **IBA1** | 0.5590 | 0.0056 | ** | 0.7341 | 0.0001 | *** | -0.3687 | 0.0834 | ns | -0.2236 | 0.3052 | ns |
| **PDGFRβ** | 0.8341 | < 0.00001 | **** | 0.6812 | 0.0003 | *** | 0.0730 | 0.7408 | ns | 0.2528 | 0.2446 | ns |
| **UAE-Lectin** | 0.3364 | 0.1166 | ns | 0.4012 | 0.0578 | ns | -0.1088 | 0.6211 | ns | 0.1522 | 0.4880 | ns |
| **OATP1A2** |  |  |  | 0.6061 | 0.0022 | ** | 0.1686 | 0.4420 | ns | 0.1140 | 0.6044 | ns |
| **OATP2B1** | 0.6061 | 0.0022 | ** |  |  |  | -0.0507 | 0.8182 | ns | -0.1292 | 0.5568 | ns |
| **OATP1C1** | 0.1686 | 0.4420 | ns | -0.0507 | 0.8182 | ns |  |  |  | 0.6604 | 0.0006 | *** |
| **OATP4A1** | 0.1140 | 0.6044 | ns | -0.1292 | 0.5568 | ns | 0.6604 | 0.0006 | *** |  |  |  |
|  |  |  |  |  |  |  |  |  |  |  |  |  |

Supplementary Table S5 Correlation analysis of integrated intensity of markers of interest with GFAP, IBA1, PDGFRβ, UAE-lectin, and each OATP isoform (OATP1A2, OATP2B1, OATP1C1, OATP4A1 in non-tumour (epilepsy) neurosurgical tissue.

| **Marker of Interest** | **OATP1A2** | | | **OATP2B1** | | | **OATP1C1** | | | **OATP4A1** | | |
| --- | --- | --- | --- | --- | --- | --- | --- | --- | --- | --- | --- | --- |
|  | **r** | **P value** | **Summary** | **r** | **P value** | **Summary** | **r** | **P value** | **Summary** | **r** | **P value** | **Summary** |
| **GFAP** | 0.8929 | 0.2667 | ns | 0.3214 | 0.4976 | ns | -0.0714 | 0.9063 | ns | 0.0919 | 1.0000 | ns |
| **IBA1** | 0.0357 | 0.9635 | ns | 0.2500 | 0.5560 | ns | -0.4643 | 0.3024 | ns | -0.5714 | 0.2000 | ns |
| **PDGFRβ** | 0.2857 | 0.5560 | ns | 0.6429 | 0.1389 | ns | -0.4286 | 0.3536 | ns | -0.2500 | 0.5948 | ns |
| **UAE-Lectin** | -0.2500 | 0.5948 | ns | -0.2857 | 0.5560 | ns | -0.5714 | 0.2000 | ns | 0.1429 | 0.7825 | ns |
| **OATP1A2** |  |  |  | 0.8292 | 0.0123 | * | 0.5714 | 0.2000 | ns | 0.1786 | 0.7131 | ns |
| **OATP2B1** | 0.8929 | 0.0123 | * |  |  |  | 0.3571 | 0.4444 | ns | -0.0714 | 0.9063 | ns |
| **OATP1C1** | 0.5714 | 0.2000 | ns | 0.3571 | 0.4444 | ns |  |  |  | 0.1071 | 0.8397 | ns |
| **OATP4A1** | 0.1786 | 0.7131 | ns | -0.0714 | 0.9063 | ns | 0.1071 | 0.8397 | ns |  |  |  |

References

1. Gao BO, Hagenbuch B, Kullak-Ublick GA, Benke D, Aguzzi A, Meier PJ. Organic Anion-Transporting Polypeptides Mediate Transport of Opioid Peptides across Blood-Brain Barrier 1. Published online 2000. Accessed September 14, 2021. http://www.jpet.org

2. W L, H G, LH S, et al. Polymorphisms in human organic anion-transporting polypeptide 1A2 (OATP1A2): implications for altered drug disposition and central nervous system drug entry. *J Biol Chem*. 2005;280(10):9610-9617. doi:10.1074/JBC.M411092200

3. H G, DG B, GK D, et al. Intestinal drug transporter expression and the impact of grapefruit juice in humans. *Clin Pharmacol Ther*. 2007;81(3):362-370. doi:10.1038/SJ.CLPT.6100056

4. Y M, T S, K K, et al. Expression of the steroid and xenobiotic receptor and its possible target gene, organic anion transporting polypeptide-A, in human breast carcinoma. *Cancer Res*. 2006;66(1):535-542. doi:10.1158/0008-5472.CAN-05-1070

5. H A, T N, C Y, et al. Enhanced expression of organic anion transporting polypeptides (OATPs) in androgen receptor-positive prostate cancer cells: possible role of OATP1A2 in adaptive cell growth under androgen-depleted conditions. *Biochem Pharmacol*. 2012;84(8):1070-1077. doi:10.1016/J.BCP.2012.07.026

6. R L, M S, K W, et al. Different expression patterns of organic anion transporting polypeptides in osteosarcomas, bone metastases and aneurysmal bone cysts. *Oncol Rep*. 2009;22(6):1485-1492. doi:10.3892/OR_00000591

7. MR B, MJ M, O B, F J, F GSM, JJ M. Expression of transporters potentially involved in the targeting of cytostatic bile acid derivatives to colon cancer and polyps. *Biochem Pharmacol*. 2006;72(6):729-738. doi:10.1016/J.BCP.2006.06.007

8. J K, Y C, AT N, D K. A novel human organic anion transporting polypeptide localized to the basolateral hepatocyte membrane. *Am J Physiol Gastrointest Liver Physiol*. 2000;278(1). doi:10.1152/AJPGI.2000.278.1.G156

9. M S, K W, B T, et al. Expression of organic anion-transporting polypeptides 1B1 and 1B3 in ovarian cancer cells: relevance for paclitaxel transport. *Biomedicine & pharmacotherapy = Biomedecine & pharmacotherapie*. 2011;65(6):417-426. doi:10.1016/J.BIOPHA.2011.04.031

10. Buxhofer-Ausch V, Secky L, Wlcek K, et al. Tumor-Specific Expression of Organic Anion-Transporting Polypeptides: Transporters as Novel Targets for Cancer Therapy. *Journal of Drug Delivery*. 2013;2013:1-12. doi:10.1155/2013/863539

11. Y C, J K, AT N, et al. Detection of the human organic anion transporters SLC21A6 (OATP2) and SLC21A8 (OATP8) in liver and hepatocellular carcinoma. *Lab Invest*. 2003;83(4):527-538. doi:10.1097/01.LAB.0000065015.02412.48

12. Abe T, Unno M, Onogawa T, et al. LST-2, A human liver-specific organic anion transporter, determines methotrexate sensitivity in gastrointestinal cancers. *Gastroenterology*. 2001;120(7):1689-1699. doi:10.1053/GAST.2001.24804

13. J K, Y C, AT N, D K. Localization and genomic organization of a new hepatocellular organic anion transporting polypeptide. *J Biol Chem*. 2000;275(30):23161-23168. doi:10.1074/JBC.M001448200

14. N T, K K, ER J, et al. A cancer-specific variant of the SLCO1B3 gene encodes a novel human organic anion transporting polypeptide 1B3 (OATP1B3) localized mainly in the cytoplasm of colon and pancreatic cancer cells. *Mol Pharm*. 2013;10(1):406-416. doi:10.1021/MP3005353

15. M N, T F, S M, et al. Identification of a new organic anion transporting polypeptide 1B3 mRNA isoform primarily expressed in human cancerous tissues and cells. *Biochem Biophys Res Commun*. 2012;418(4):818-823. doi:10.1016/J.BBRC.2012.01.115

16. NR M, S L, Y X, H Y, AS B, JA M. Potent cytotoxicity of the phosphatase inhibitor microcystin LR and microcystin analogues in OATP1B1- and OATP1B3-expressing HeLa cells. *Mol Cancer Ther*. 2007;6(2):587-598. doi:10.1158/1535-7163.MCT-06-0500

17. Hamada A, Sissung T, Price DK, et al. Effect of SLCO1B3 haplotype on testosterone transport and clinical outcome in Caucasian patients with androgen-independent prostatic cancer. *Clin Cancer Res*. 2008;14(11):3312. doi:10.1158/1078-0432.CCR-07-4118

18. JL W, EM K, EA O, et al. Expression of SLCO transport genes in castration-resistant prostate cancer and impact of genetic variation in SLCO1B3 and SLCO2B1 on prostate cancer outcomes. *Cancer Epidemiol Biomarkers Prev*. 2011;20(4):619-627. doi:10.1158/1055-9965.EPI-10-1023

19. M M, T O, T S, et al. Human liver-specific organic anion transporter-2 is a potent prognostic factor for human breast carcinoma. *Cancer Sci*. 2007;98(10):1570-1576. doi:10.1111/J.1349-7006.2007.00570.X

20. SR V, D J, M F, U G, PJ M, GA KU. The human organic anion transporting polypeptide 8 (SLCO1B3) gene is transcriptionally repressed by hepatocyte nuclear factor 3beta in hepatocellular carcinoma. *J Hepatol*. 2004;40(2):212-218. doi:10.1016/J.JHEP.2003.10.008

21. B G, RD H, A W, et al. Localization of organic anion transporting polypeptides in the rat and human ciliary body epithelium. *Exp Eye Res*. 2005;80(1):61-72. doi:10.1016/J.EXER.2004.08.013

22. VL S. Prostaglandin transport. *Prostaglandins Other Lipid Mediat*. 2002;68-69:633-647. doi:10.1016/S0090-6980(02)00061-8

23. K W, M S, T T, F S, G K, W J. Altered expression of organic anion transporter polypeptide (OATP) genes in human breast carcinoma. *Cancer Biol Ther*. 2008;7(9):1450-1455. doi:10.4161/CBT.7.9.6282

24. K W, M S, J R, et al. The analysis of organic anion transporting polypeptide (OATP) mRNA and protein patterns in primary and metastatic liver cancer. *Cancer Biol Ther*. 2011;11(9):801-811. doi:10.4161/CBT.11.9.15176

25. VR H, MG B, P Y, RA N, RN D. Regulation of prostaglandin transporters in colorectal neoplasia. *Cancer Prev Res (Phila)*. 2008;1(2):93-99. doi:10.1158/1940-6207.CAPR-07-0009

26. Bronger H, König J, Kopplow K, et al. ABCC Drug Efflux Pumps and Organic Anion Uptake Transporters in Human Gliomas and the Blood-Tumor Barrier. Published online 2005. doi:10.1158/0008-5472.CAN-05-1271

27. M G, K K, S O, et al. Organic anion transporting polypeptide 2B1 is a high-affinity transporter for atorvastatin and is expressed in the human heart. *Clin Pharmacol Ther*. 2006;80(6):607-620. doi:10.1016/J.CLPT.2006.09.010

28. D K, T N, K I, J N, A T, I T. Involvement of human organic anion transporting polypeptide OATP-B (SLC21A9) in pH-dependent transport across intestinal apical membrane. *J Pharmacol Exp Ther*. 2003;306(2):703-708. doi:10.1124/JPET.103.051300

29. MV SP, B H, B U, PJ M, T S. Characterization of an organic anion-transporting polypeptide (OATP-B) in human placenta. *J Clin Endocrinol Metab*. 2002;87(4):1856-1863. doi:10.1210/JCEM.87.4.8431

30. Pressler H, Sissung TM, Venzon D, Price DK, Figg WD. Expression of OATP Family Members in Hormone-Related Cancers: Potential Markers of Progression. *PLoS ONE*. 2011;6(5). doi:10.1371/JOURNAL.PONE.0020372

31. Swanson MEV, Scotter EL, Smyth LCD, et al. Identification of a dysfunctional microglial population in human Alzheimer’s disease cortex using novel single-cell histology image analysis. *Acta Neuropathologica Communications*. 2020;8(1):1-16. doi:10.1186/S40478-020-01047-9/FIGURES/6

32. Woolf Z, Swanson ME v, Smyth LC, et al. Single-cell image analysis reveals a protective role for microglia in glioblastoma. *Neuro-oncology Advances*. 2021;3(1):1-14. doi:10.1093/NOAJNL/VDAB031

33. Rustenhoven J, Smyth LC, Jansson D, et al. Modelling physiological and pathological conditions to study pericyte biology in brain function and dysfunction. *BMC Neuroscience*. 2018;19(1):1-15. doi:10.1186/S12868-018-0405-4/FIGURES/7
